# Pharmacological Network Analysis of the Functions and Mechanism of Quercetin From Jisuikang (JSK) in Spinal Cord Injury (SCI)

**DOI:** 10.1111/jcmm.70269

**Published:** 2024-12-16

**Authors:** Lini Dong, Haoyu He, Zejun Chen, Xiaoxiao Wang, Yunchao Li, Guohua Lü, Bing Wang, Lei Kuang

**Affiliations:** ^1^ Department of Geriatrics, The Second Xiangya Hospital Central South University Changsha Hunan China; ^2^ Department of Spinal Surgery, The Second Xiangya Hospital Central South University Changsha Hunan China

**Keywords:** Huangqi, IL1R1, Jisuikang, microglia, quercetin, spinal cord injury

## Abstract

Neuroinflammation, especially microglia/macrophage activation, is a hallmark of spinal cord injury (SCI). Jisuikang (JSK) is a clinical experiential Chinese herbal formula for SCI therapy containing Huangqi (*Astragali Radix*), Danggui (*Angelica sinensis Radix*), Chishao (*Paeoniae Radix Rubra*), Dilong (earthworm, *Pheretima aspergillum*), Chuanxiong (*Chuanxiong Rhizoma*), Taoren (*Persicae Seman*) and Honghua (*Carthami Flos*). Eighteen active ingredients in 6 herbs of JSK were found to be correlated with inflammation, spinal injury and other diseases. These 18 active ingredients target 5464 genes according to the PubChem database. Through comparing differentially expressed genes between SCI and normal samples using GSE datasets, 50 hub genes were identified. These hub‐genes were enriched in oxidative stress response and inflammation response. The herb‐compound‐target, herb‐compound‐signalling and compound‐target‐signalling networks were generated and quercetin was identified as the hub compound. A concentration of 25 μM quercetin showed no cytotoxicity but significantly protected microglial cells from LPS‐induced inhibition of cell viability. LPS stimulation elevated the levels of iNOS, IL‐1β and TNF‐α but decreased IL‐10 levels, whereas quercetin significantly attenuated LPS‐induced alterations in these factors. Moreover, quercetin targeted gene, IL1R1 was reduced by quercetin as predicted. Overexpression of IL1R1 further increased LPS‐induced inflammation, which could be partly reversed by quercetin treatment. In vivo, quercetin improved histopathological alterations, inflammation and promoted M2 macrophage polarisation post‐injury, whereas IL1R1 overexpression partially attenuated the beneficial effects of quercetin on the rat SCI model. Collectively, quercetin, the main ingredient compound of JSK, protects against LPS‐induced cell viability inhibition and cellular inflammation, which could be partially attenuated by IL1R1 overexpression.

## 1 Introduction

Microglia are the resident macrophages in the brain and spinal cord that exert a central effect on initiating and perpetuating the immune response [1, 2, 3, 4]. Neuroinflammation, especially microglia/macrophage activation, is a hallmark of spinal cord injury (SCI) [2, 3, 4]. These cells are well known to have two activation states after pathogenic stimulation that lead to either a pro‐ or an anti‐inflammatory phenotype, known as ‘M1 and M2’, respectively, depending on the inflammatory environment [5]. For instance, proinflammatory M1 macrophages induced in vitro by a combination of IFNγ + LPS or IFNγ + tumour necrosis factor α (TNF‐α) have promoted phagocytosis function, produce proinflammatory cytokines (IL‐1β, IL‐12) and release elevated amounts of reactive oxygen species (ROS). On the contrary, anti‐inflammatory M2 macrophages induced by IL‐4 or IL‐13 produce anti‐inflammatory cytokines (such as IL‐10) and growth factors, show low production of oxidant molecules and nitric oxide and enhance the capacity of stem cells to proliferate and to differentiate [6]. TNF‐α has been recognised as one of the critical mediators for activating proinflammatory macrophages in SCI [7]. In addition, IL‐4‐deficient knock‐out aggravates inflammation and promotes M1‐like activation following spinal cord injury in mice [8]. Thus, targeting deregulated microglia or macrophage activation during SCI might be an efficient strategy for SCI treatment regimens.

Traditional Chinese medicine has been proven to be an efficient option for SCI treatment regimens during the past long period [9, 10, 11]. Jisuikang (JSK) is an herbal formula composed of Huangqi (*Astragali Radix*, 30 g), Danggui (*Angelica sinensis Radix*, 12 g), Chishao (*Paeoniae Radix Rubra*, 12 g), Dilong (earthworm, *Pheretima aspergillum*, 10 g), Chuanxiong (*Chuanxiong Rhizoma*, 10 g), Taoren (*Persicae Seman*, 10 g) and Honghua (*Carthami Flos*, 10 g). It has been reported to inhibit the expression level of nitric oxide synthase, decrease the levels of nitric oxide and TNF‐α, reduce the activity of superoxide dismutase, protect neurons, inhibit ROCK signal pathway at SCI lesion locations, finally improve motor function of rat model [12, 13]. Among the ingredients, Huangqi, a perennial herbaceous plant of the Leguminosae family, is the most commonly used single herb for SCI treatment regimens [14, 15, 16]. Another formula Danggui Sini decoction involving Danggui can alleviate chronic constriction injury and diabetes‐induced neuropathic pain [17]. However, the key active ingredients of these herbs' protection against SCI and the underlying mechanism remain unclear.

As gene expression profiles develop, bio‐informatics comprehensive analysis is the most frequently used method to search critical biomarkers for various disorders [18, 19]. Up to now, a network‐based pharmacological approach, which intends to systematically reflect and reveal the biological foundation of complex diseases with an integrated multicomponent network‐based insight, has emerged as a novel research topic [20, 21]. The vast majority of traditional Chinese medicine (TCM) and global ethnic medicine target various molecules in the human body to produce healing efficacy [22, 23, 24]. Network pharmacology is a new strategy based on the theory of systems biology, network analysis of biological systems and the selection of specific signal nodes for multi‐target drug molecule design [20]. Several network pharmacology‐related research have been used in investigating the potential mechanism of TCM formulas in SCI treatment, including Buyang Huanwu Decoction and Yaobitong capsule [25, 26] Thus, the holistic approaches of network‐based pharmacology in TCM research might be practical choices for treating spinal cord injury.

Traditional Chinese Medicine System Pharmacology Database (TCMSP, http://lsp.nwu.edu.cn/tcmsp.php) [27], an accessible systems pharmacology platform of Chinese herbal medicines that captures and predicts the relationships between drugs, targets and diseases, is built on the basis of TCM literature and scientific publications, containing over 13,731 pure ingredients extracted from 505 TCM herbs. Twelve important properties like human oral bioavailability (OB), half‐life (HL), drug‐likeness (DL), Caco‐2 permeability (Caco‐2) and blood–brain barrier (BBB) are provided for drug screening and evaluation. In the present study, we retrieved the main active compounds of herbs in JSK from TCMSP, analysed the targets of the main active compounds and SCI, employed Gene Expression Omnibus (GEO) expression profiles to analyse differentially expressed genes between SCI and control samples and identified overlapped genes as hub‐genes. Active ingredients‐targets‐signalling pathway networks were constructed, and quercetin was selected for further experiments. Lipopolysaccharide (LPS) is commonly used in research to induce inflammation, serving as a model to simulate the inflammatory processes that occur in spinal cord injury (SCI) [28]. By using LPS, this study aims to mimic the inflammatory environment of SCI, thereby investigating the potential therapeutic effects of JSK and quercetin in reducing SCI‐related inflammation. The specific effects of quercetin on LPS‐simulated microglial cells were examined, and the involvement of IL1R1 in quercetin protection against LPS‐induced cellular damage was investigated.

## 2 Materials and Methods

### 2.1 Retrieval of Compound Information in JSK

The chemical ingredients of Huangqi, Danggui, Chishao, Chuanxiong, Taoren and Honghua were collected using the Traditional Chinese Medicine System Pharmacology Database (TCMSP, http://lsp.nwu.edu.cn/tcmsp.php) [27]. Screening of the active compounds was performed based on ADME (absorption, distribution, metabolism and excretion), and using pharmacokinetic information retrieval filters, bio‐active ingredients were retrieved for further analysis under screening conditions of OB ≥ 30% [29] and DL ≥ 0.2 [30].

### 2.2 Compound Targets and Disease Target Retrieval

The protein targets of the active compounds in Huangqi, Danggui, Chishao, Chuanqiong, Taoren and Honghua were obtained from the PubChem database (https://pubchem.ncbi.nlm.nih.gov/). The disease target genes of SCI were obtained through analysing differentially expressed genes between SCI and normal samples.

### 2.3 Differentially Expressed Genes Between SCI and Normal Spinal Cord Tissue Samples

Two publicly available datasets, GSE42828 and GSE5296, were used in this study, and each dataset was designed with different experimental aims. The GSE5296 dataset (96 samples) focused on a comprehensive time‐course study of SCI in mice. In this dataset, the SCI model group consisted of mice subjected to moderate contusion injury at the T8 spinal cord segment. Samples were collected at multiple time points (0.5, 4, 24 and 72 h, as well as 7 and 28 days) from both the lesion site and adjacent spinal cord regions (rostral and caudal). Additionally, sham samples were collected at the same time points and regions, where the mice underwent a laminectomy without spinal cord injury, serving as pseudo‐injury controls. Six uninjured (naïve) mice samples were also included. In the present study, 54 injured samples, 36 sham samples expression profiles were analysed.

The GSE42828 dataset focused on the early molecular responses to SCI and included spinal cord samples from wild‐type and Trkb.T1 knockout mice. For the purposes of our analysis, only wild‐type samples were considered. Samples were collected from SCI mice at 1, 3 and 7 days post‐injury, with a control group consisting of uninjured wild‐type mice. In total, this dataset included 13 SCI samples and 4 control samples. This dataset provided insight into the early molecular changes following SCI, complementing the longer time‐course data from GSE5296.

Individual *p*‐values were computed, and the false discovery rate (FDR) for multiple testing corrections was calculated using the Benjamini and Hochberg methods. Differentially expressed genes (DEGs) were considered using the threshold of |logFC| > 0.6 and adj.*p*.Val < 0.05.

### 2.4 Protein–Protein Interaction (PPI) Network Analysis

To further analyse the 231 DEGs, a PPI network was constructed using the STRING database (version 12.0, https://string‐db.org/). A combined interaction score greater than 0.7 was applied as the threshold to identify significant protein interactions. The resulting PPI network was then visualised using Cytoscape software (version 3.10.2, https://cytoscape.org/). In order to identify key hub genes within the PPI network, the Molecular Complex Detection (MCC) plugin in Cytoscape was utilised. The top 50 hub genes were identified based on their interaction scores.

### 2.5 GO, KEGG and WikiPathways Analysis

Gene ontology (GO) functional enrichment analysis was performed using the Metascape tool (https://metascape.org/gp/) [31]. The analysis focused on identifying significantly enriched GO terms related to biological processes. GO terms with thresholds of Count ≥ 2 and EASE (Expression Analysis Systematic Explorer) scores ≤ 0.05 were selected for functional annotation clustering. Additionally, 12 hub genes were identified from the PPI network constructed using the STRING database. The Molecular Complex Detection (MCC) plugin in Cytoscape was used to identify the top 50 hub genes, of which the top 12 were selected for further analysis. KEGG (Kyoto Encyclopedia of Genes and Genomes) pathway enrichment analysis was also conducted using the Metascape tool. In addition to GO and KEGG analysis, the DEGs were further analysed using WikiPathways (https://www.wikipathways.org/).

### 2.6 Construction and Analysis of Network Pharmacology

To comprehensively understand the molecular mechanisms of JSK for SCI, Cytoscape software was used to construct the compound‐target‐pathway networks [32]. In these graphical networks, the compounds, proteins, or pathways were represented by nodes, while the compound‐target or target‐pathway interactions were represented by edges.

### 2.7 The Binding Ability Between Quercetin and Spinal Injury‐Related Target Genes Predicted by Molecular Docking

The 3D crystal structure of quercetin was downloaded from the PubChem (http://pubchem.ncbi.nlm.nih.gov/compound/). The 3D crystal structure of the 11 spinal injury‐related target genes (CDK1, FOS, CXCL1, ICAM1, IL1R1, CXCL10, MYC, PTGS2, RB1, CCL2 and TLR4) was downloaded from the Protein Data Bank (PDB) (http://www.rcsb.org/pdb/). The AutoDock Vina [33] (https://vina.scripps.edu/) was used for the semi‐flexible docking of quercetin and proteins.

### 2.8 Cell Lineage and LPS Stimulation

Mouse microglial cell line (BV2) was procured from Procell (China) and cultured in MEM/high glucose containing 10% FBS and 1% penicillin/streptomycin. For the induction of the cellular inflammation model, 1 μg/mL LPS was added to microglia for 24 h.

### 2.9 Determination of the Maximum Non‐Cytotoxic Concentration of Quercetin

Microglial cells were cultured for 24 h at 1 × 10^4^ cells/well in 96‐well plates, followed by treatment for 12 h with gradient doses of quercetin (0, 1, 10, 25, 50, 50, 100 and 200 μM). MTT assays were subsequently performed to determine cell viability, and the maximum non‐cytotoxic concentration of quercetin was selected.

### 2.10 MTT Assay Detecting Cell Viability

Following treatment with gradient doses of quercetin or co‐treatment with gradient doses of quercetin and LPS, 5 mg/mL MTT solution (3‐(4,5‐Dimethylthiazol‐2‐yl)‐2,5‐diphenyltetrazolium bromide; Sigma, USA) was added (10 μL per well); next, cells were incubated for another 4 h at 37°C. The supernatants were subsequently removed, and DMSO was added (100 μL per well). A Multiskan MS microplate reader (Labsystems, Finland) was used to measure the absorbance at 490 nm.

### 2.11 qRT‐PCR

Using TRIzol reagent, total RNA was isolated from spinal cord tissue samples as directed by the manufacturer's instructions, and a RevertAid First Strand cDNA Synthesis Kit (Thermo Fisher Scientific, Waltham, MA, USA) was used to reversely transcribe extracted RNA into cDNA. PCR‐based analyses were conducted on an ABI 7500 Real Time PCR system with SYBR Green qPCR Mix (Beyotime). Relative expression levels were calculated by taking GAPDH as an internal reference.

### 2.12 ELISA

After transduction and/or stimulation, iNOS, IL‐1β, TNF‐α and IL‐10 concentrations in the culture medium were examined with corresponding enzyme‐linked immunosorbent assay (ELISA) kit (Abcam and CUSABIO, China) following the instruction of the manufactures.

### 2.13 Immunoblotting

The proteins were isolated from target cells using RIPA buffer as previously described [34]. After electrophoresis with SDS‐PAGE, the separated proteins were transferred onto PVDF (polyvinylidene difluoride) membrane and subsequently identified by immunoblotting using the indicated primary antibodies. Antibodies against iNOS (ab15323, Abcam, Cambridge, UK), IL‐1β (ab254360, Abcam), TNF‐α (ab183218, Abcam), IL‐10 (ab133575, Abcam), p38 (14064‐1‐AP, Proteintech, Wuhan, China), p‐p38 (AF4001, Affinity, Changzhou, China), ERK1/2 (67170‐1‐Ig, Proteintech), p‐ERK1/2 (sc‐81492, Santa Cruz, Dallas, TX, USA), JNK (AF6319, Affinity), p‐JNK (AF3318, Affinity), p65 (10745‐1‐AP, Proteintech), p‐p65 (ab194726, Abcam) and β‐actin (60009‐1‐Ig, Proteintech) were used. Membranes were cultured using corresponding horseradish peroxidase‐conjugated antibodies against rabbit or mouse IgG (Abcam). Thermo Scientific Pierce ECL Western Blotting Substrate was used to visualise the protein bands.

### 2.14 Immunofluorescent Staining (IF Staining) Confirming Microglia Polarisation

Cells were seeded in 6‐well plates with a coverslip placed first. For staining, cells were fixed for 10 min using 4% paraformaldehyde in cold PBS, then washed three times with 2% PBS‐BSA (bovine serum albumin). Cells were incubated overnight at 4°C with primary antibodies diluted 1:100 in 2% PBS‐BSA: anti‐CD11b and anti‐iNOS (Abcam). After washing three times with 2% PBS‐BSA, cells were incubated for 1 h with the secondary antibody, using cy3‐conjugated anti‐rabbit IgG antibody and FITC‐conjugated anti‐mouse IgG antibody (Beyotime) at a 1/500 dilution. Nuclei were stained with DAPI (1 μg/mL; Sigma‐Aldrich). Following three additional PBS washes, the cells were observed under a fluorescent microscope (Olympus).

### 2.15 Lentivirus Transduction

For IL1R1 overexpression, a recombinant lentivirus that overexpresses mouse IL1R1 was purchased from Genechem (Shanghai, China) and infected microglial cells following the protocols. 48 h later, IL1R1 overexpression was confirmed using qRT‐PCR and immunoblotting.

### 2.16 A Rat Model of SCI and Quercetin and JSK Treatment

Male Sprague–Dawley rats weighing 200–250 g were acquired from Hunan Silaike Jingda Laboratory Animal Co. Ltd. (Changsha, China). The experimental procedures were conducted following the guidelines recommended by the Animal Care and Use Committee of The Second Xiangya Hospital of Central South University with approval from the same committee. To investigate the role of IL‐1R1 in the treatment of quercetin, the study involved 30 rats, which were evenly divided into five experimental groups: a Sham group, an SCI group, an SCI + quercetin group, an SCI + quercetin + lv‐NC group and an SCI + quercetin + lv‐IL1R1 group. The Sham group underwent only a laminectomy, while the SCI group received an intraperitoneal injection (i.p.) of the vehicle (1 mL sterile saline plus 1% DMSO) following SCI. Based on prior research indicating that the duration of quercetin treatment post‐SCI enhances functional recovery [35], the SCI + quercetin group was treated with 7.5 mg/kg quercetin dissolved in 1 mL of the vehicle, administered i.p. twice daily for 14 days post‐SCI. Following SCI, 10 μL of lentivirus (about 10^6^ TU) containing the rat IL1R1‐overexpressing segment was injected in situ. The SCI group was given the same dose of an empty lentiviral vector. Behavioural test was performed at 0, 1, 3, 7, 10 and 14 days post‐SCI. To compare the therapeutic efficacy of JSK and QUE, 24 rats were divided into 4 groups: a Sham group, an SCI group, an SCI + JSK group, SCI + quercetin group. For the SCI + JSK group, rats were treated with JSK at 50 g/kg/day and administered intragastrically as previously described [12]. The rest of the groups' administrations were performed as above mentioned. Behavioural test was performed at 0, 1, 3, 7, 10, 14, 21 and 28 days post‐SCI.

Anaesthesia was induced using sodium pentobarbital (30 mg/kg). A bilateral laminectomy at the T8 vertebrae was performed to expose the spinal cord, and a spinal cord crush injury was inflicted using the MASCIS Impactor device. An impact of a onto the exposed spinal cord Manual bladder expression was carried out daily until the rats regained their micturition reflex.

### 2.17 Behavioural Tests

The Basso‐Beattie‐Bresnahan (BBB) scoring system was employed to evaluate the open‐field locomotor activity of rats. Assessments were conducted one day prior to and at 0, 1, 3, 7, 10 and 14 days post‐SCI, adhering to the BBB methodology [36]. This scoring system assigns functional scores from 0 to 21, assessing movement of the ankle, hip, knee and trunk, along with the coordination of each rat. Locomotor performance was monitored and documented by two independent evaluators who were unaware of the experimental group assignments.

### 2.18 Tissue Preparation and Haematoxylin & Eosin (H&E) Staining

At specific time points, each rat was sedated, and a segment of the spinal cord measuring 5 mm both above and below the T8 injury site was swiftly excised, placed in a cryotube, flash‐frozen in liquid nitrogen and stored at −80°C. Subsequently, the spinal tissue adjacent to the T8 injury site (5 mm above and below) was excised, immersed in paraformaldehyde overnight and prepared for further analysis.

Tissue samples were dehydrated using a series of xylene and graded alcohol solutions, embedded in paraffin and sectioned into 5‐μm slices using a microtome. After regular dewaxing and rehydration, sections were stained with a HE staining kit (Beyotime) according to the manufacturer's instructions.

### 2.19 Tissue Sample IF Staining

After regular dewaxing and rehydration, the sections were subjected to antigen retrieval in EDTA buffer for 15 min at 95°C. The sections were then blocked with 10% goat serum (Boster Biological Technology Ltd) for 30 min at 37°C and further were incubated overnight with primary antibodies against CD68, IBA1, Arg1, iNOS, NeuN and IL1R1 (Abcam) at 4°C. After washing with PBS three times for 5 min, the sections were incubated with FITC or cy3 labelled secondary antibodies (dilution 1:500, Beyotime) for 1 h at 37°C, then washed with PBS. Nuclei were stained with DAPI (1 μg/mL; Sigma‐Aldrich) for 5 min, followed by washing and sealing with an anti‐fluorescence quencher. Images were captured using a fluorescence microscope (Olympus). The fluorescence density was determined by ImageJ (NIH, USA).

### 2.20 Statistical Analysis

Experimental data from individual experiments were presented as the mean ± standard deviation (SD). Using GraphPad Prism Software, comparisons between conditions were performed by Student's *t*‐test or one‐way ANOVA with Tukey's test post hoc analysis. A *p* value greater than 0.05 means that no effect was observed.

## 3 Results

### 3.1 Retrieval of Active Ingredients of 6 Herbs in JSK and Drug Target Genes

**FIGURE 1:**
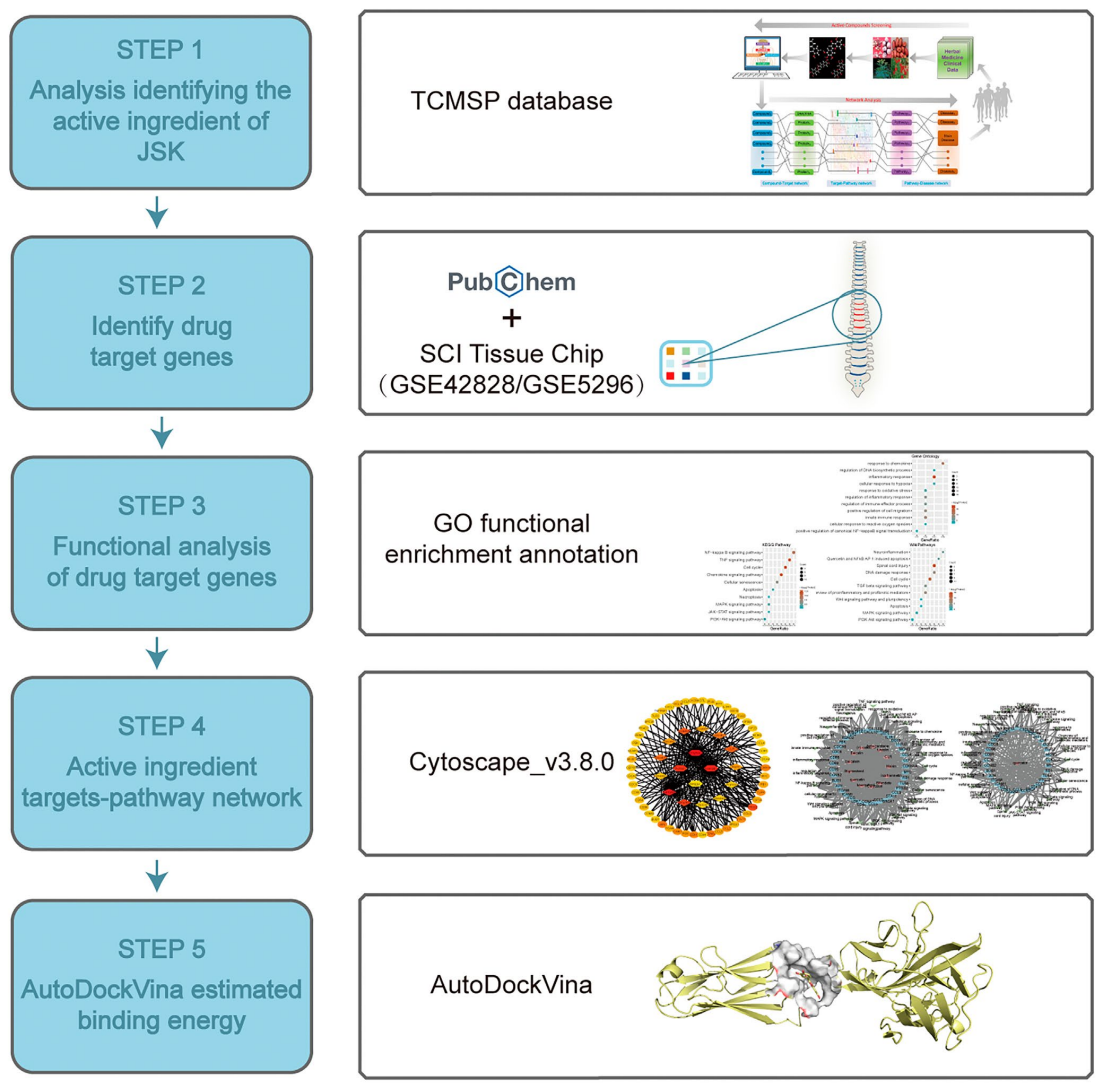
Pharmacological network analysis of the functions and mechanism of Jisuikang (JSK) in spinal cord injury (SCI).

The flow chart of active ingredients in JSK and drug target genes selection was shown in Figure 1. The main active ingredients of Huangqi, Danggui, Chishao, Chuanxiong, Taoren and Honghua were analysed by the online Chinese medicine database TCMSP; a total of 18 active ingredients were obtained from other 6 herbs using the threshold of OB ≥ 30% and DL ≥ 0.2 and are shown in Table 1.

**TABLE 1:** Main active ingredients in herbs of JSK according to TCMSP database.

TCM	Name	Mol ID	MW	OB (%)	DL	Target
Huangqi	Mairin	MOL000211	456.78	55.37707338	0.7761	61
	Isorhamnetin	MOL000354	316.28	49.60437705	0.306	60
	Bifendate	MOL000387	418.38	31.09782391	0.66553	21
	Formononetin	MOL000392	268.28	69.67388061	0.21202	54
	Calycosin	MOL000417	284.28	47.75182783	0.24278	57
	Kaempferol	MOL000422	286.25	41.88224954	0.24066	243
	Quercetin	MOL000098	302.25	46.43334812	0.27525	4459
Danggui	Beta‐sitosterol	MOL000358	414.79	36.91390583	0.75123	1
	Stigmasterol	MOL000449	412.77	43.82985158	0.75665	45
Chishao	Baicalein	MOL002714	270.25	33.51891869	0.20888	125
	Baicalin	MOL002776	446.39	40.12360996	0.75264	43
	Beta‐sitosterol	MOL000358	414.79	36.91390583	0.75123	1
	Sitosterol	MOL000359	414.79	36.91390583	0.7512	1
	Stigmasterol	MOL000449	412.77	43.82985158	0.75665	45
	(+)‐catechin	MOL000492	290.29	54.82643405	0.24164	1030
Chuanxiong	Sitosterol	MOL000359	414.79	36.91390583	0.7512	1
Taoren	Beta‐sitosterol	MOL000358	414.79	36.91390583	0.75123	1
Honghua	Phytoene	MOL002706	545.04	39.56307142	0.50463	2
	Baicalein	MOL002714	270.25	33.51891869	0.20888	125
	Quercetagetin	MOL002721	318.25	45.00699322	0.30991	9
	Beta‐carotene	MOL002773	536.96	37.18433337	0.58358	183
	Baicalin	MOL002776	446.39	40.12360996	0.75264	43
	Beta‐sitosterol	MOL000358	414.79	36.91390583	0.75123	1
	Kaempferol	MOL000422	286.25	41.88224954	0.24066	243
	Stigmasterol	MOL000449	412.77	43.82985158	0.75665	45
	Luteolin	MOL000006	286.25	36.16262934	0.24552	265
	CLR	MOL000953	386.73	37.87389754	0.67677	338
	Quercetin	MOL000098	302.25	46.43334812	0.27525	4459

**FIGURE 2:**
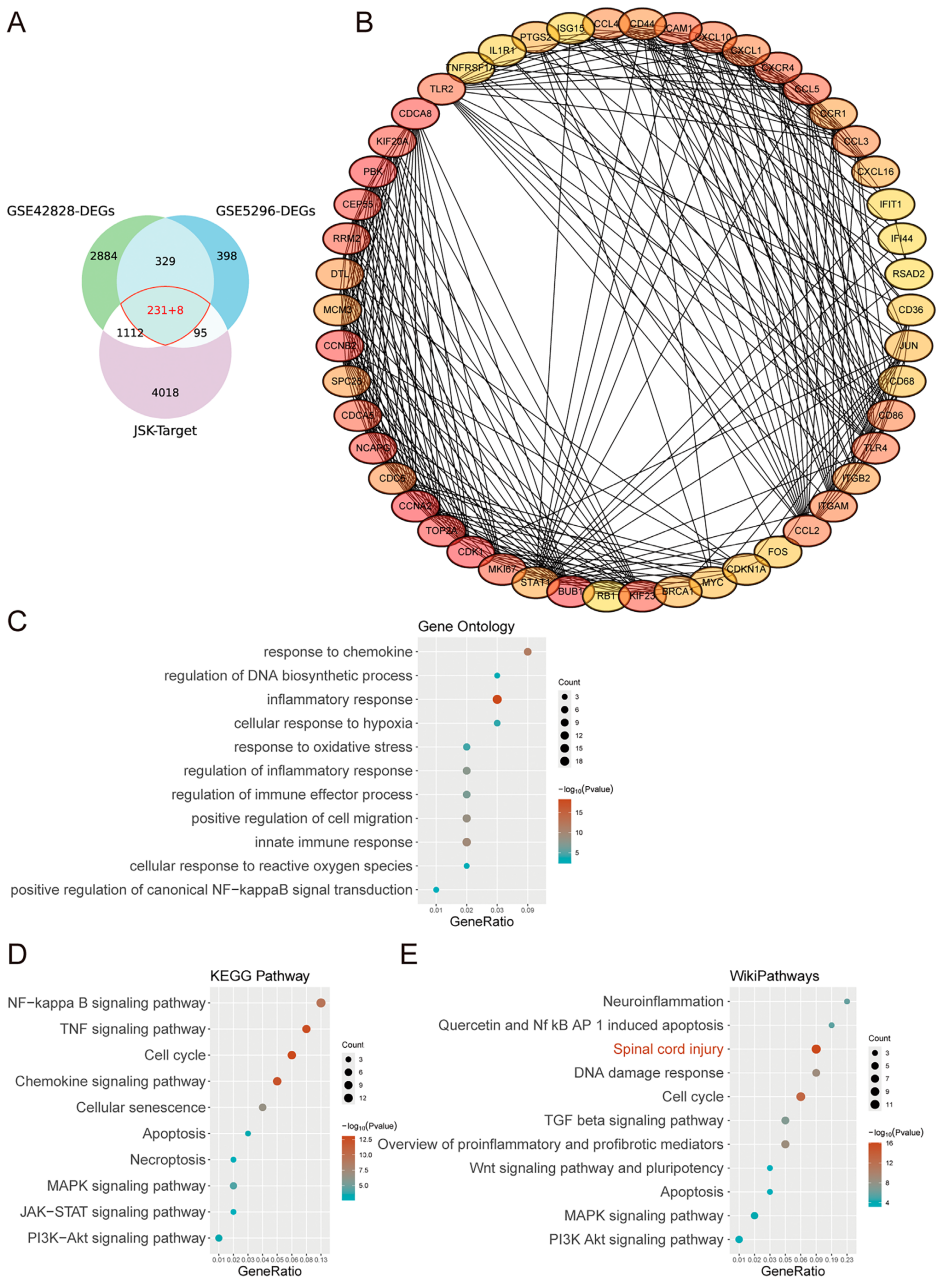
Integrate network pharmacology and pharmacological analysis identifying the active ingredient of Jisuikang (JSK). (A) The chemical compounds of 6 herbs in JSK were retrieved from Traditional Chinese Medicine System Pharmacology Database (TCMSP, http://lsp.nwu.edu.cn/tcmsp.php). Differentially expressed genes between spinal cord injury samples and normal control samples were obtained based on GSE42828 and GSE5296. The intersection of these sets highlights 239 overlapping genes. Among them, 231 genes were consistently differentially expressed in the two GSE sets. (B) Protein–protein interaction (PPI) network of the 231 DEGs shows significant interactions between the target genes identified using the STRING database with a confidence score > 0.7. The nodes represent target genes, and the edges represent the interactions between them. (C) Gene Ontology (GO) enrichment analysis of the 231 DEGs, indicating processes such as inflammatory response, DNA biosynthetic process regulation, and chemokine responses. (D) KEGG pathway enrichment analysis shows significant pathways like NF‐kappa B signalling, TNF signalling, and apoptosis. (E) WikiPathways enrichment analysis displaying key pathways involved in spinal cord injury and apoptosis.

A total of 5464 drug target genes of these 18 active ingredients were retrieved from PubChem (Figure 2A). Differentially expressed genes between spinal cord injury samples and control samples were obtained based on GSE42828 (4564 DEGs) and GSE5296 (1061 DEGs) (Figure S1A,B). The intersection of three gene sets revealed 231 genes that were consistently differentially expressed in both datasets and overlapped with drug target genes (Figure 2A). These 231 DEGs were further analysed using the STRING database to construct a protein–protein interaction (PPI) network, and only targets with an interaction score greater than 0.7 were selected. The network was visualised using Cytoscape, and the top 50 hub genes were identified using the MCC plugin (Figure 2B). Functional enrichment analysis was conducted using Metascape, revealing significant involvement of the DEGs in several biological processes, including inflammatory response, chemokine‐mediated signalling, innate immune response, positive regulation of cell migration, regulation of DNA biosynthetic processes, oxidative stress response, reactive oxygen species response, immune effector regulation, hypoxia response, inflammatory response regulation and positive regulation of NF‐κB signalling (Figure 2C). KEGG pathway enrichment analysis showed that the DEGs were involved in pathways such as chemokine signalling, TNF signalling, cell cycle, cellular senescence, MAPK signalling, apoptosis, PI3K‐Akt signalling, JAK–STAT signalling and necroptosis (Figure 2D). WikiPathways analysis further highlighted the involvement of these DEGs in pathways associated with pro‐inflammatory and pro‐fibrotic mediators, spinal cord injury, cell cycle, DNA damage response, TGF‐β signalling, neuroinflammation, MAPK signalling, apoptosis, PI3K‐Akt signalling, Wnt signalling and quercetin‐induced apoptosis via NF‐κB and AP‐1 (Figure 2E).

**FIGURE 3:**
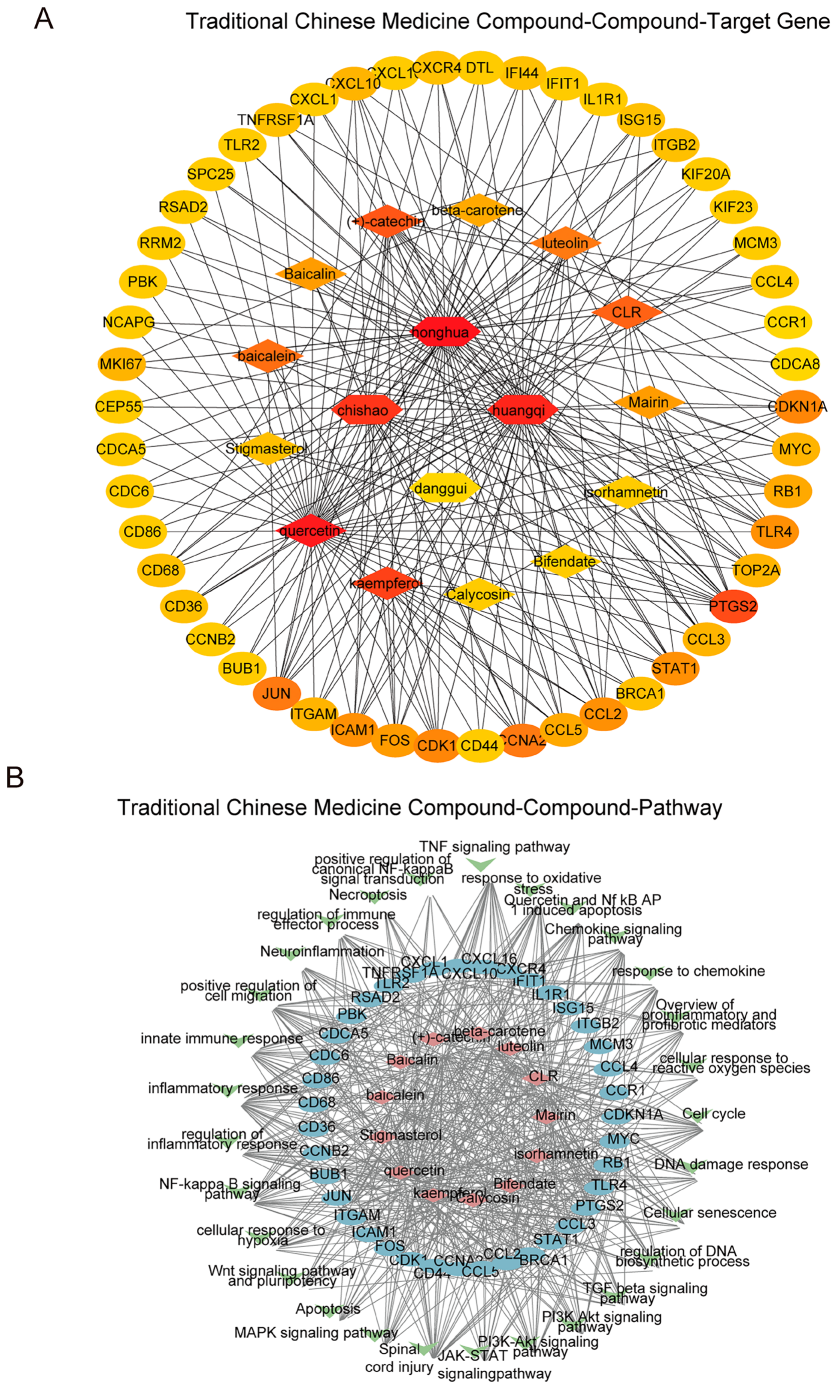
Network consists of herbals, active compounds, and differentially expressed drug target genes generated by the Cytoscape plugin MCC. (A) The herb‐compound‐target gene network is based on traditional Chinese medicine (TCM) compounds from JSK. The herbs are represented in the center, compounds in the middle, and target genes on the outer ring. (B) Herb‐compound‐target signalling pathway network. The nodes represent signalling pathways, and the edges represent the interactions between compounds and pathways.

**FIGURE 4:**
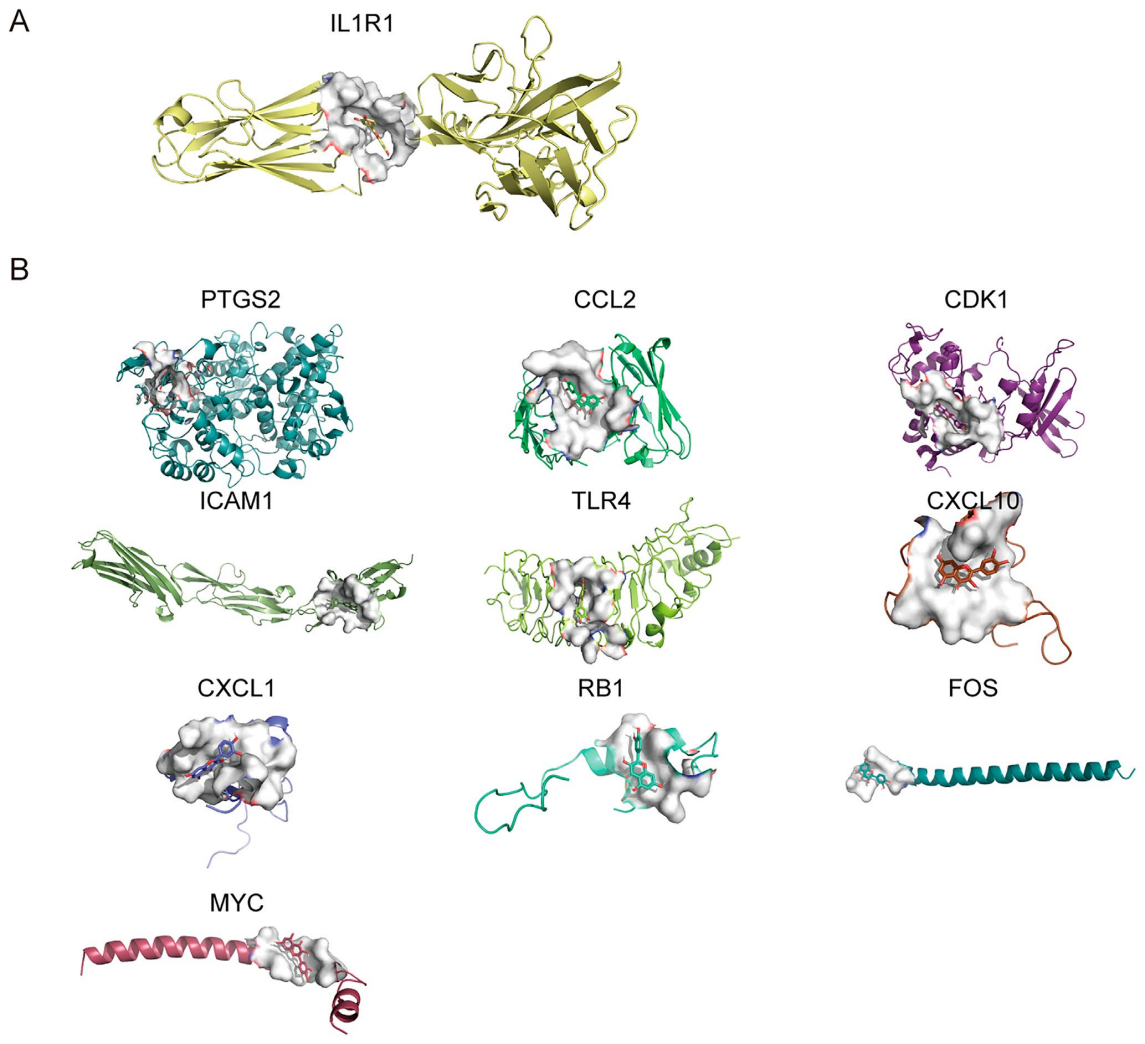
Molecular docking. (A and B) Docking results of quercetin with key targets involved in SCI, including IL1R1 (A), CDK1, FOS, CXCL1, ICAM1, CXCL10, MYC, PTGS2, RB1, CCL2, and TLR4. The docking scores (in kcal/mol) represent the binding affinity between quercetin and the respective target proteins, with lower scores indicating stronger binding interactions.

To comprehensively understand the molecular mechanisms of the 50 hub‐genes upon SCI, Cytoscape software was used to construct the herb‐compound‐target (Figure 3A) and compound‐target‐signalling (Figure 3B). The herb‐compound‐target network was constructed using Cytoscape to map the relationships between 50 differentially expressed drug target genes, their related active compounds and the herbs from JSK. The MCC plugin in Cytoscape was used to identify the top active compounds based on their degree scores. Among the 13 compounds, quercetin was found to have the highest MCC score, indicating that it has the most interactions with the identified target genes. The network contains a total of 67 nodes and 255 edges, reflecting the complex interactions between the herbs, active compounds and their target genes (Figure 3A). The herb‐compound‐signalling pathway network was constructed to display the relationships between active compounds, their target genes and the signalling pathways they influence. This herb‐compound‐target‐signalling pathway network includes 81 nodes and 568 edges, highlighting the involvement of quercetin and other active compounds in key signalling pathways such as the NF‐κB signalling pathway, PI3K‐Akt signalling pathway and TNF signalling pathway, which are crucial in regulating inflammation and cell survival following spinal cord injury (Figure 3B). The quercetin‐target and signalling pathway network included 66 nodes and 251 edges, further supporting quercetin's central role in modulating these biological processes (Figure S2). Key targets involved in SCI, such as CDK1, FOS, CXCL1, ICAM1, IL1R1, CXCL10, MYC, PTGS2, RB1, CCL2 and TLR4, were identified as being significantly upregulated in SCI (Table 2). Protein structures of these targets were downloaded from the PDB database, and quercetin's 3D structure was obtained from PubChem. Docking simulations were performed using AutoDock Vina to evaluate the binding interactions between quercetin and these targets (Figure 4, Table 3). Among the identified targets, the binding affinity of IL1R1 is −6.786 kcal/mol and it has been reported to play a critical role in inflammatory pathways in SCI [37, 38]; thus, IL1R1 was selected for further experimental validation.

**TABLE 2:** SCI‐related drug/disease target genes differentially expressed in SCI samples.

Gene	Data	logFC	Reg	*p*
CCL2	GSE5296	1.73305829	Up	1.54E‐05
CCL2	GSE42828	5.32	Up	6.10E‐05
CDK1	GSE5296	0.69091976	Up	0.0153
CDK1	GSE42828	3.57	Up	1.39E‐05
CXCL1	GSE5296	1.37159112	Up	0.0038
CXCL1	GSE42828	1.65	Up	0.0339
CXCL10	GSE5296	1.33867625	Up	2.88E‐07
CXCL10	GSE42828	2.58	Up	0.000222
FOS	GSE5296	0.88245231	Up	4.56E‐05
FOS	GSE42828	2.12	Up	8.46E‐05
ICAM1	GSE5296	1.11796456	Up	2.13E‐09
ICAM1	GSE42828	3.86	Up	9.25E‐12
IL1R1	GSE5296	0.60815019	Up	0.00731
IL1R1	GSE42828	3.68	Up	5.94E‐09
MYC	GSE5296	1.07110641	Up	3.06E‐06
MYC	GSE42828	2.96	Up	4.88E‐06
PTGS2	GSE5296	1.04866892	Up	0.00368
PTGS2	GSE42828	4.08	Up	0.000401
RB1	GSE5296	0.67810506	Up	0.0498
RB1	GSE42828	0.702	Up	5.15E‐05
TLR4	GSE5296	0.68567858	Up	0.000249

**TABLE 3:** The molecular docking of quercetin and the protein ofs SCI‐related drug/disease target genes.

Target	Score (kcal/mol)
PTGS2	−9.676
CCL2	−7.587
CDK1	−7.573
ICAM1	−6.867
IL1R1	−6.786
TLR4	−6.675
CXCL10	−6.396
CXCL1	−6.011
RB1	−5.447
FOS	−5.398
MYC	−5.341

### 3.2 Maximum Non‐Cytotoxic Concentration of Quercetin

**FIGURE 5:**
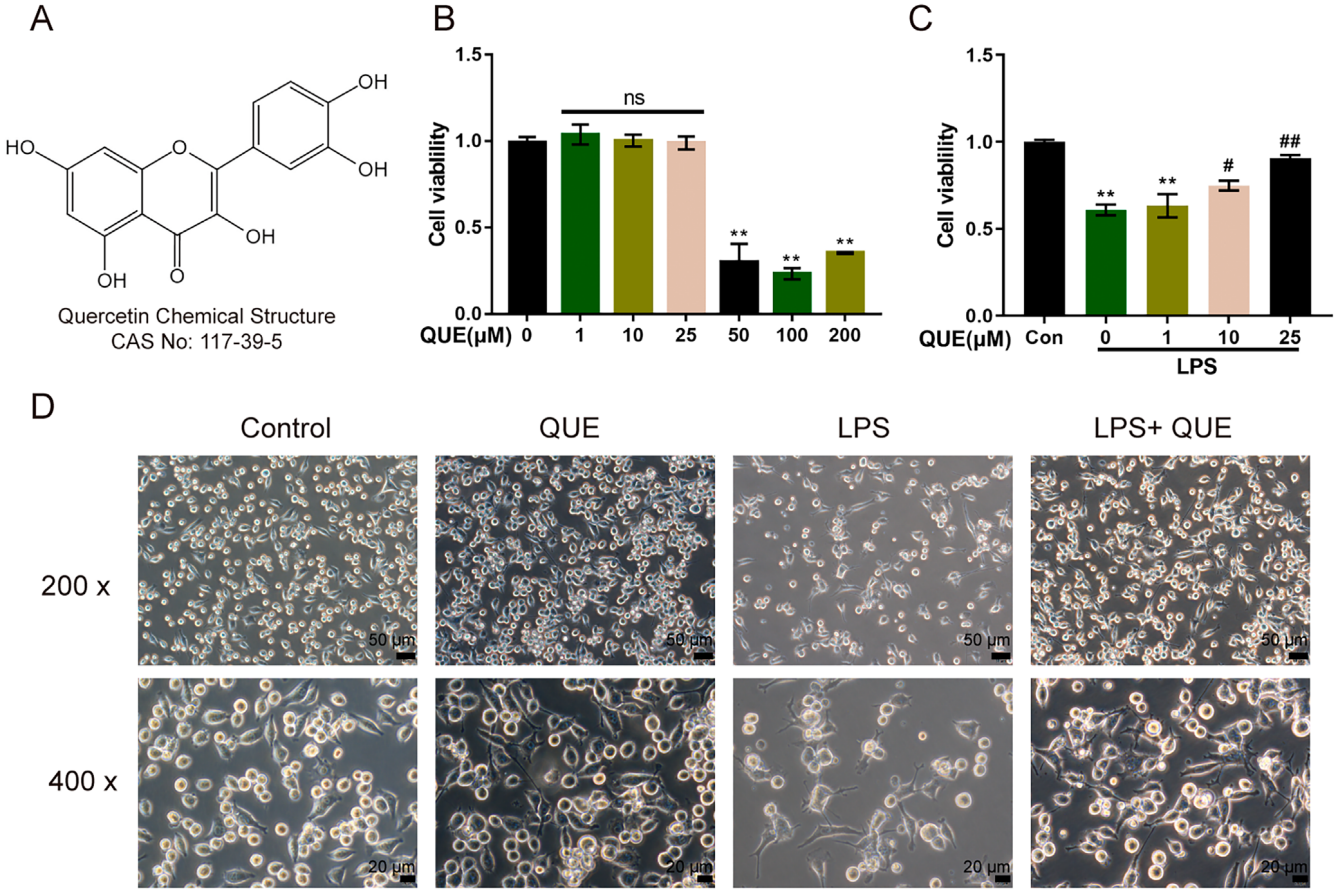
Maximum non‐cytotoxic concentration of quercetin. (A) The chemical structure of quercetin. (B) Microglial cells were treated with gradient concentrations of quercetin (0, 1, 10, 25, 50, 100, and 200 μM) for 12 h and examined for cell viability by MTT assay. **p < 0.01 represents a comparison with the 0 μM group. (C) Microglial cells were pre‐treated with gradient concentrations of quercetin (0, 1, 10, 25 μM) for 12 h followed by the treatment of 1 μg/mL LPS for 24 h; cell viability was determined using MTT assay. *p < 0.05, **p < 0.01 represents a comparison of the LPS group with the Con group. ^#^ *p* < 0.05, ^##^ *p* < 0.01, represents a comparison with the LPS group. (D) Representative images of microglial cells under quercetin and/or LPS treatment (200× and 400×). *n* = 3.

Figure 5A demonstrated the chemical structure of quercetin. For determining the maximum non‐cytotoxic concentration of quercetin, we stimulated microglial cells with gradient concentrations of quercetin (0, 1, 10, 25, 50, 100 and 200 μM) for 12 h and performed MTT assay to examine cell viability; Figure 5B shows that 1, 10 or 25 μM quercetin had no cytotoxicity on microglial cells. For quercetin protection against LPS‐induced cellular inflammation, we pre‐treated microglial cells for 12 h with gradient concentrations of quercetin (0, 1, 10 and 25 μM), and then treated these cells for 24 h with 1 μg/mL LPS; we performed MTT assay to determine cell viability. Figure 5C showed that LPS significantly inhibited cell viability, whereas 10 and 25 μM quercetin significantly increased microglial cell viability. And 25 μM quercetin exhibited a better effect. Given these findings, 25 μM quercetin was selected for further experiments. Representative images of microglial cells under quercetin and/or LPS treatment were shown in Figure 5D.

### 3.3 Quercetin Improves LPS‐Induced Cellular Inflammation in Microglial Cells

**FIGURE 6:**
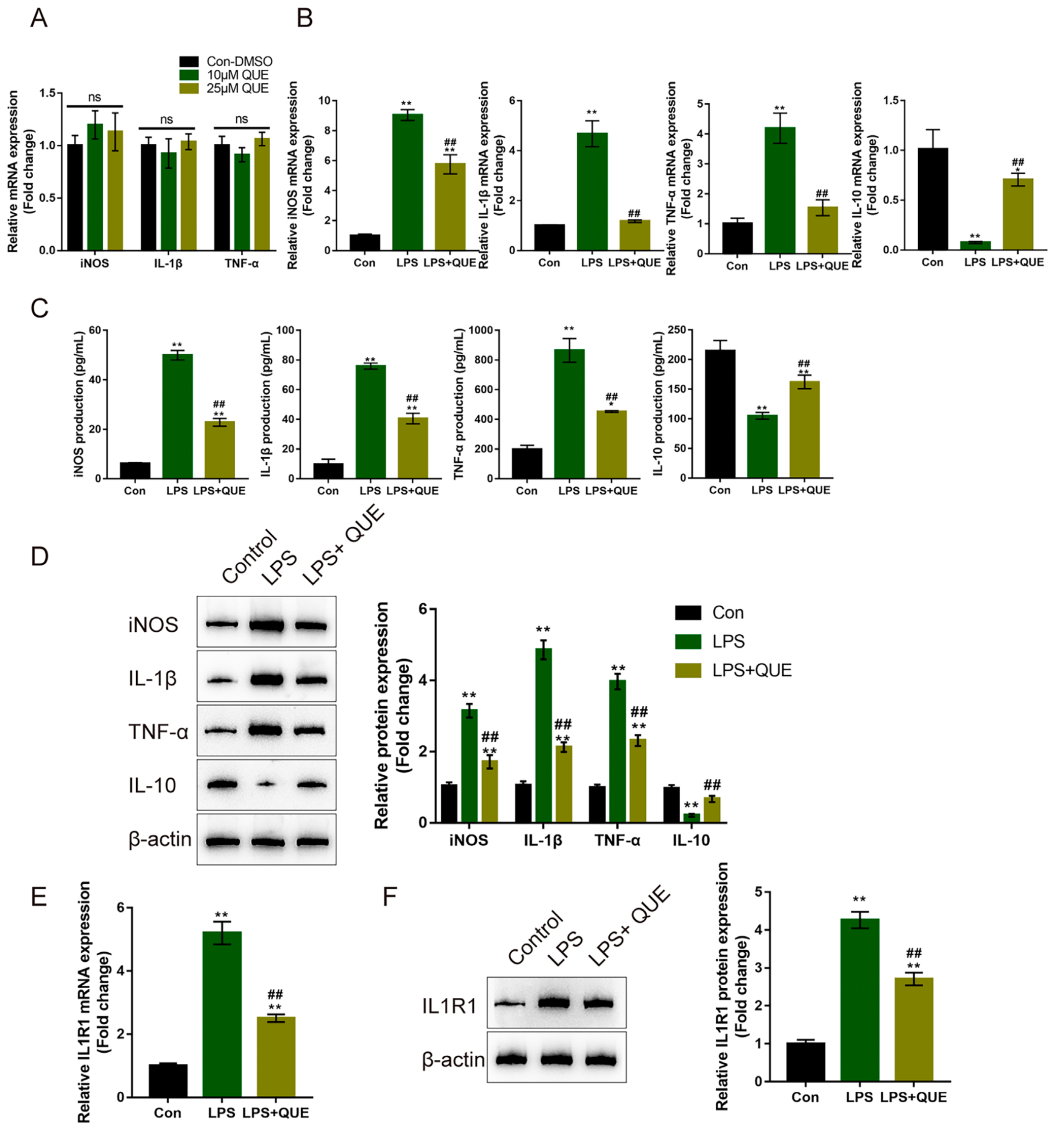
Quercetin improves LPS‐induced cellular inflammation in microglial cells. (A) Microglial cells were non‐treated or treated with 10 μM or 25 μM quercetin and examined for the mRNA expression of iNOS, IL‐1β, and TNF‐α using qRT‐PCR. *n* = 3. (B–E) Microglial cells were subsequently non‐treated or pre‐treated with 25 μM quercetin followed by the treatment of 1 μg/mL LPS and examined for the mRNA expression of iNOS, IL‐1β, TNF‐α, and IL‐10 using qRT‐PCR (B); the production of iNOS, IL‐1β, TNF‐α, and IL‐10 using ELISA (C); the protein levels of iNOS, IL‐1β, TNF‐α, and IL‐10 using Immunoblotting (D); the mRNA expression of IL1R1 using qRT‐PCR (E); the protein levels of IL1R1 by Immunoblotting (F). *n* = 3, *p < 0.05, **p < 0.01 represents a comparison of the LPS group with the Con group or a comparison of the LPS + QUE group with the Con group. ^##^ *p* < 0.01 represents a comparison of the LPS + QUE group with the LPS group.

The inflammatory response in SCI is characterised by predominant and prolonged M1 macrophages/microglia polarisation. Inhibition of the M1 and promotion of M2 phenotype are potentially more feasible approaches for controlling neuroinflammation in SCI [2, 39, 40]. Thus, microglial cells were non‐treated or treated with 10 or 25 μM quercetin and examined for the M1 and M2 phenotype microglial cells secreted cytokines iNOS, IL‐1β, TNF‐α and IL‐10 mRNA expression; Figure 6A shows that single quercetin treatment caused no alterations in the mRNA expression of iNOS, IL‐1β, TNF‐α and IL‐10. However, under LPS stimulation, the mRNA expression, culture medium concentrations, and iNOS, IL‐1β and TNF‐α protein contents were significantly induced but IL‐10 was decreased; 25 μM quercetin partially eliminated LPS‐induced alterations in iNOS, IL‐1β, TNF‐α and IL‐10 (Figure 6B–D). The mRNA expression and protein levels of IL1R1 were also investigated; Figure 6E,F shows that LPS significantly induced IL1R1 levels, whereas 25 μM quercetin partially abolished the promotive effects of LPS on IL1R1.

### 3.4 Quercetin Modulates Microglia M1/M2 Polarisation

**FIGURE 7:**
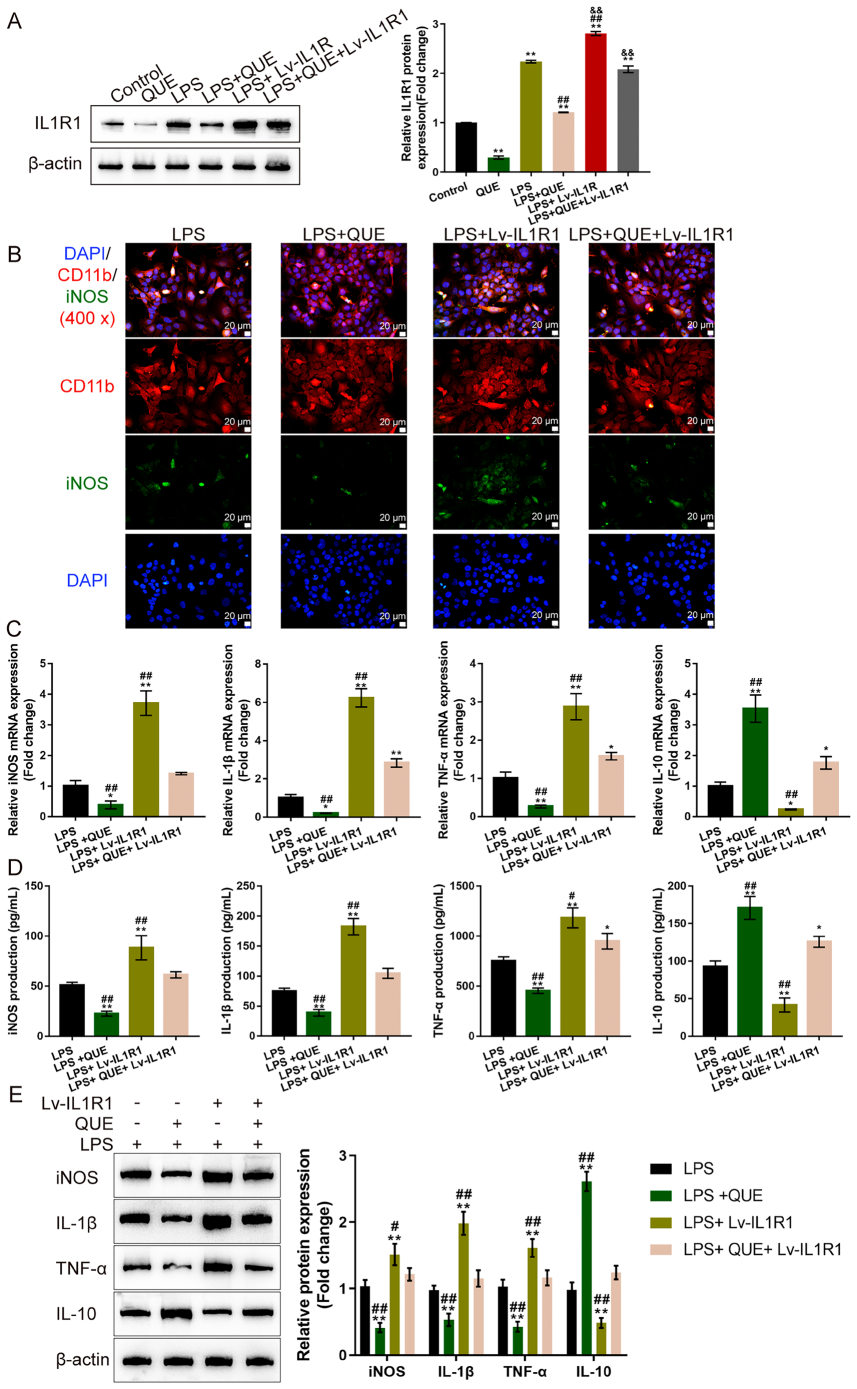
Quercetin modulates microglia M1/M2 polarisation. (A) Cells were divided into control, QUE, LPS, LPS + QUE, LPS + Lv‐IL1R1, and LPS + QUE + Lv‐IL1R1 groups. IL1R1 overexpression was determined using Immunoblotting. (*n* = 3) **p < 0.01 represents a comparison with the control group. ^##^ *p* < 0.01 represents a comparison with the LPS group. ^&&^ *p* < 0.01 represents a comparison with the LPS + QUE group. Next, microglia cells were divided into four groups, LPS, LPS + QUE, LPS + Lv‐IL1R1, and LPS + QUE + Lv‐IL1R1, and treated and transduced accordingly. (B) The levels of CD11b and iNOS in cells was examined using Immunofluorescent staining (IF staining). (C) The mRNA expression of IL‐1β, TNF‐α, iNOS, and IL‐10 was examined using qRT‐PCR; (D) The production of IL‐1β, TNF‐α, iNOS, and IL‐10 were examined using ELISA; (E) The protein levels of IL‐1β, TNF‐α, iNOS, and IL‐10 were examined using Immunoblotting; *n* = 3. *p < 0.05, **p < 0.01 represents a comparison with the LPS group. ^#^ *p* < 0.05, ^##^ *p* < 0.01 represents a comparison with the LPS + QUE + Lv‐IL1R1 group.

Regarding the underlying mechanism, IL1R1 overexpression was achieved in microglia cells by introducing IL1R1‐overexpressing lentivirus (Lv‐IL1R1); the transduction efficiency was verified using the qRT‐PCR and immunoblotting (Figure S3). IL1R1 protein levels were examined under quercetin treatment with or without LPS stimulation. Figure 7A shows that without LPS stimulation, quercetin significantly decreased the protein levels of IL1R1. LPS remarkably elevated IL1R1 protein levels, which were enhanced by IL1R1 transfection but attenuated by quercetin treatment (Figure 7A). Next, microglia cells were divided into four groups, LPS, LPS + QUE, LPS + Lv‐IL1R1 and LPS+ QUE+ Lv‐IL1R1, and treated and transduced accordingly. As for the activation of microglia (M1 polarisation), the contents of CD11b (Microglia marker) and iNOS (M1 polarisation marker) were determined using IF staining. Figure 7B shows that CD11b saw no significant differences among groups. Quercetin remarkably decreased, whereas IL1R1 overexpression increased the intensity of iNOS; the promotive effects of IL1R1 overexpression on iNOS content were partially attenuated by quercetin (Figure 7B). Quercetin treatment significantly decreased the mRNA expression (Figure 7C), production (Figure 7D) and protein levels of IL‐1β, TNF‐α and iNOS (Figure 7E) while increasing IL‐10 mRNA, production and protein level (Figure 7C–E). IL1R1 overexpression, however, dramatically increased the mRNA expression (Figure 7C), production (Figure 7D), and protein levels of IL‐1β, TNF‐α and iNOS (Figure 7E) while decreasing IL‐10 mRNA, production, and protein level (Figure 7C–E). Under quercetin treatment, IL1R1 overexpression‐induced changes in IL‐1β, TNF‐α, iNOS and IL‐10 mRNA, production and protein levels in LPS‐stimulated microglia were partially eliminated by quercetin treatment (Figure 7C–E).

### 3.5 The NF‐κB and MAPK Signalling Pathways Are Involved in the Process of Quercetin‐Modulating Microglia M1/M2 Polarisation

**FIGURE 8:**
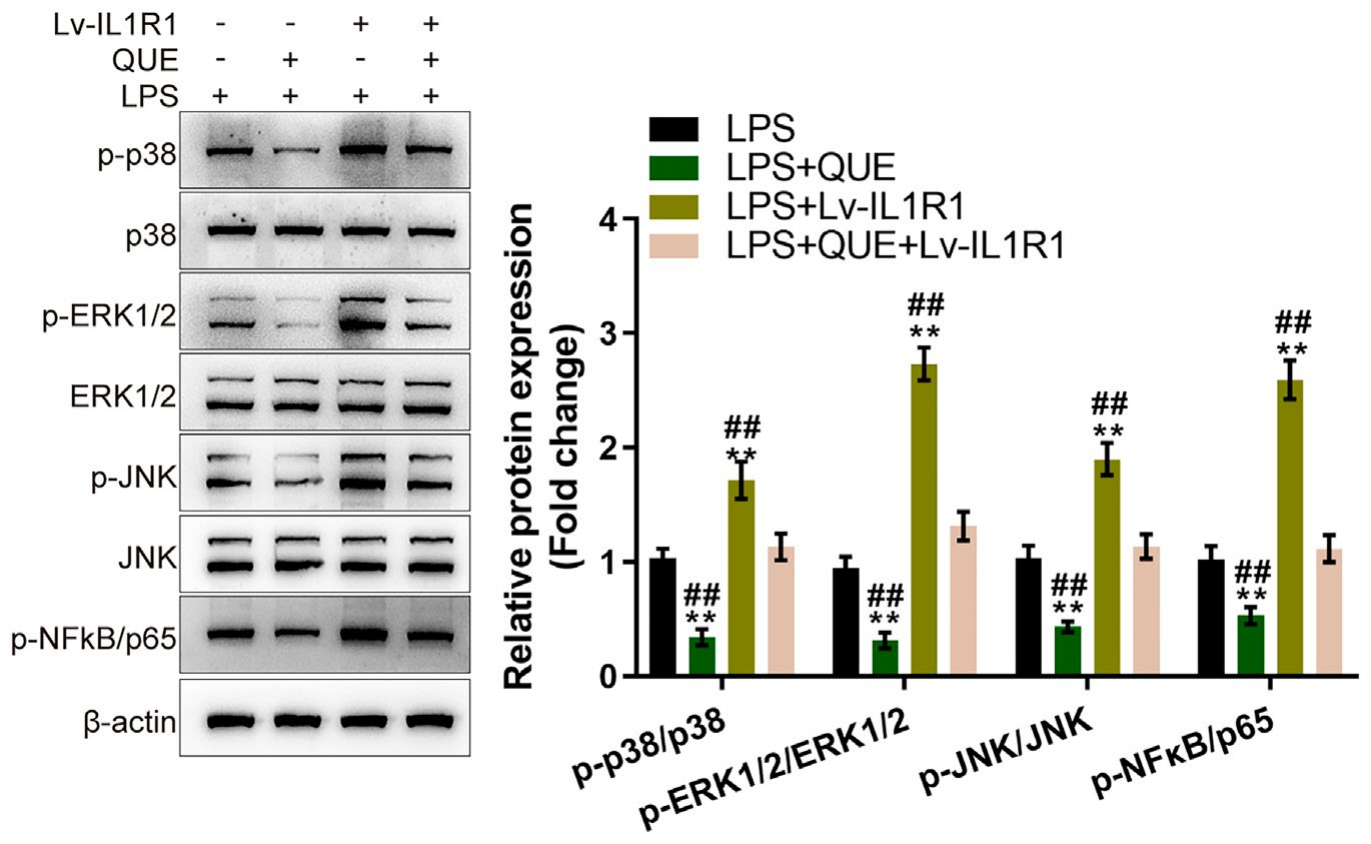
The NF‐κB and MAPK signalling pathways are involved in the process of quercetin modulating microglia M1/M2 polarisation. Microglia cells were divided into four groups, LPS, LPS + QUE, LPS + Lv‐IL1R1, and LPS+ QUE+ Lv‐IL1R1, and treated and transduced accordingly; the protein levels of p38, p‐p38, ERK1/2, p‐ERK1/2, JNK, p‐JNK, p65, and p‐p65 were examined using Immunoblotting. *n* = 3. **p < 0.01 represents a comparison with the LPS group. ^##^ *p* < 0.01. Represents a comparison with the LPS + QUE + Lv‐IL1R1 group.

Next, the changes in the NF‐κB and MAPK signalling, including the phosphorylation of p38, ERK1/2, JNK and p65 were also monitored. Quercetin treatment significantly inhibited the phosphorylation of p38, ERK1/2, JNK and p65 in LPS‐stimulated microglia (Figure 8). IL1R1 overexpression exerted opposite effects by promoting the phosphorylation of p38, ERK1/2, JNK and p65 in LPS‐stimulated microglia (Figure 8). Similarly, in LPS‐stimulated microglia, IL1R1 overexpression‐caused changes in p38, ERK1/2, JNK and p65 phosphorylation were significantly attenuated by quercetin treatment (Figure 8).

### 3.6 In Vivo Effects of Quercetin on a Rat SCI Model

**FIGURE 9:**
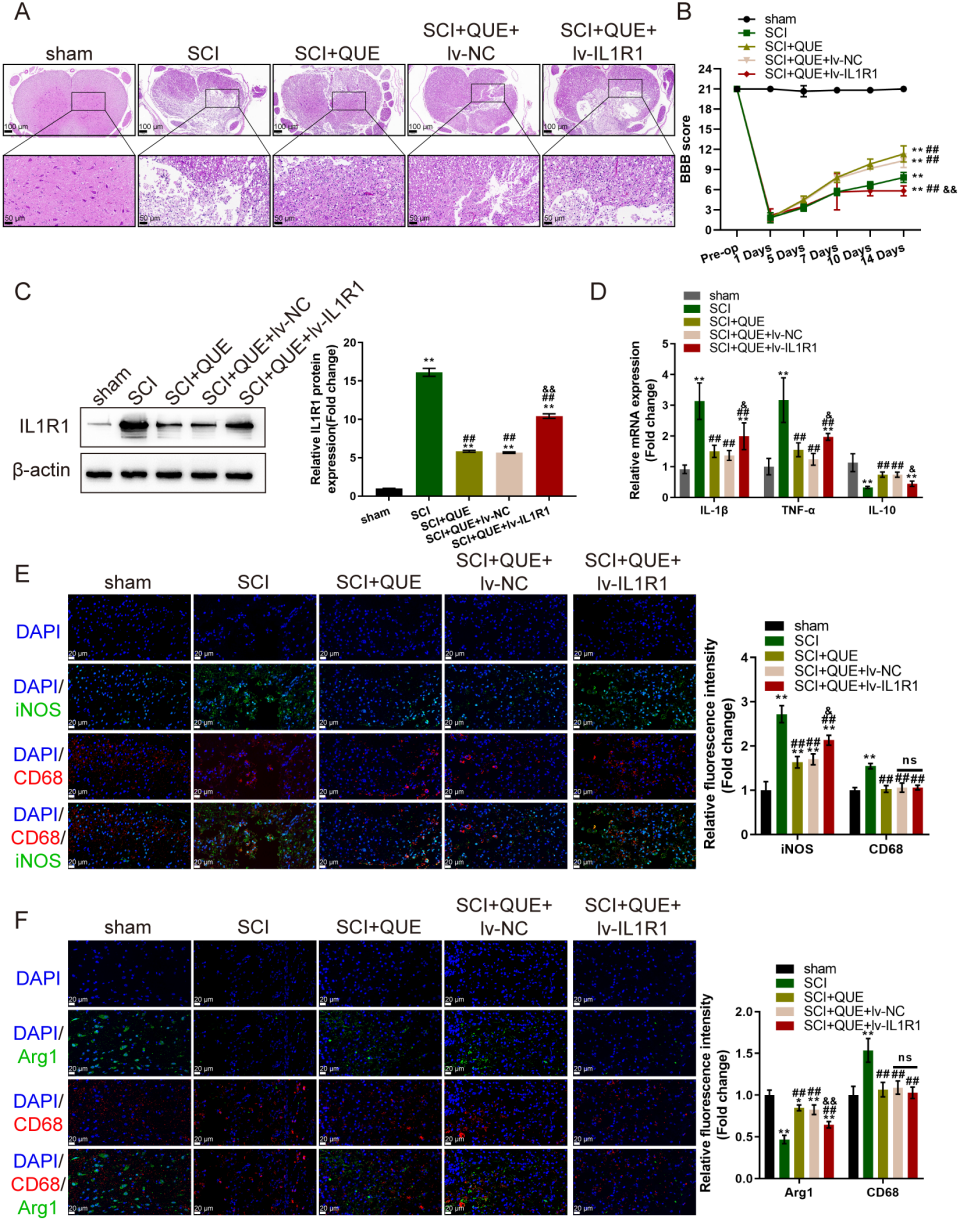
In vivo effects of quercetin on a rat SCI model. Male Sprague–Dawley rats were exploited for SCI modelling. Quercetin treatment and lentivirus overexpressing IL1R1 were conducted. (A) At day 14 post‐SCI injury, histopathological changes in the spinal cord at the site of injury were evaluated using H&E staining. (B) The Basso‐Beattie‐Bresnahan (BBB) behavioural score system was used to evaluate motor function in the hind limbs of rats in each group at 0, 1, 3, 7, 10, and 14 days post‐injury. (C) The protein levels of IL1R1 at the site of injury were evaluated using Immunoblotting. (D) At day 14 post‐SCI injury, the mRNA expression of pro‐inflammatory cytokines TNF‐α, IL‐1β, and IL‐10 was determined using qRT‐PCR. (E and F) At day 14 post‐SCI injury, M1 microglia/macrophage marker iNOS, M2 macrophage marker Arg1, and microglia/macrophage marker CD68 were detected at the rat spinal cord injury site by IF staining. *N* = 6 or 3. * *p* < 0.05, **p < 0.01 represents a comparison with the sham group. ^##^ *p* < 0.01 represents a comparison with the SCI + QUE group. ^&^ *p* < 0.05, ^&&^ *p* < 0.01 represents a comparison with the SCI + QUE + lv‐NC group.

As for the in vivo effects of quercetin, SD rats were used to establish an SCI model. On day 14 of post‐SCI injury, histopathological changes in the spinal cord at the site of injury were evaluated. Figure 9A shows that quercetin improved, whereas IL1R1 overexpression worsened the necrosis at the site of spinal cord injury. The BBB behavioural scoring indicated a sharply impaired motor function in the hind limbs of rats at 1d post‐injury; quercetin treatment significantly improved the motor function, whereas the beneficial effects were partially attenuated by IL1R1 overexpression (Figure 9B). The protein levels of IL1R1 were drastically elevated at the site of injury but partially decreased by quercetin treatment (Figure 9C). At day 14 post‐SCI injury, the mRNA expression of pro‐inflammatory cytokines TNF‐α, IL‐1β and IL‐10 were determined. Figure 9D shows that TNF‐α and IL‐1β were elevated, IL‐10 levels were decreased at the injury site but reversed by quercetin; the effects of quercetin were partially attenuated by IL1R1 overexpression. Also at day 14 post‐SCI injury, the number of microglia was increased in the SCI tissues (increased CD68 expression), M1 microglia/macrophage marker iNOS drastically elevated, whereas M2 microglia/macrophage marker Arg1 decreased at rat spinal cord injury site; quercetin decreased CD68 expression and iNOS expression but increased Arg1, whereas IL1R1 overexpression partially attenuated the effects of quercetin on Arg1 and iNOS expression (Figure 9E,F), suggesting quercetin promoted M2 polarisation and inhibited M1 polarisation which could be revised by IL‐1R1 overexpression.

In addition, the therapeutic effects of JSK and quercetin were monitored and compared over a longer recovery period after SCI. The observation period was extended to 28 days post‐SCI to evaluate long‐term functional recovery. H&E staining of spinal cord sections revealed extensive tissue damage and disruption of spinal cord architecture in the SCI group. Both JSK and quercetin treatments improved tissue integrity, with noticeable reductions in the extent of damage at 28 days post‐SCI compared to the untreated SCI group (Figure S4A). In addition, according to the BBB locomotor score, significant improvements in motor function were observed with both JSK and quercetin treatments compared to the SCI group, with recovery continuing beyond the 14‐day mark. By day 28, both treatments had resulted in similar levels of recovery, and no statistically significant difference was detected between the two treatments (Figure S4B). Then, IL1R1 expression was analysed at 28 days post‐SCI. Both treatments led to a significant downregulation of IL1R1 expression compared to the SCI group, with no notable differences between JSK and quercetin (Figure S4C). Regarding inflammatory cytokine levels (IL‐1β and IL‐10) in spinal cord tissues, both treatments were found to reduce the expression of the pro‐inflammatory cytokine IL‐1β and increase the expression of the anti‐inflammatory cytokine IL‐10. Similarly, no significant differences were observed between the effects of JSK and quercetin, suggesting that quercetin alone may be as effective as the multi‐herbal JSK formulation in modulating the inflammatory response (Figure S4D).

### 3.7 Quercetin Modulates Microglial Polarisation and IL1R1 Expression in an SCI Rat Model

**FIGURE 10:**
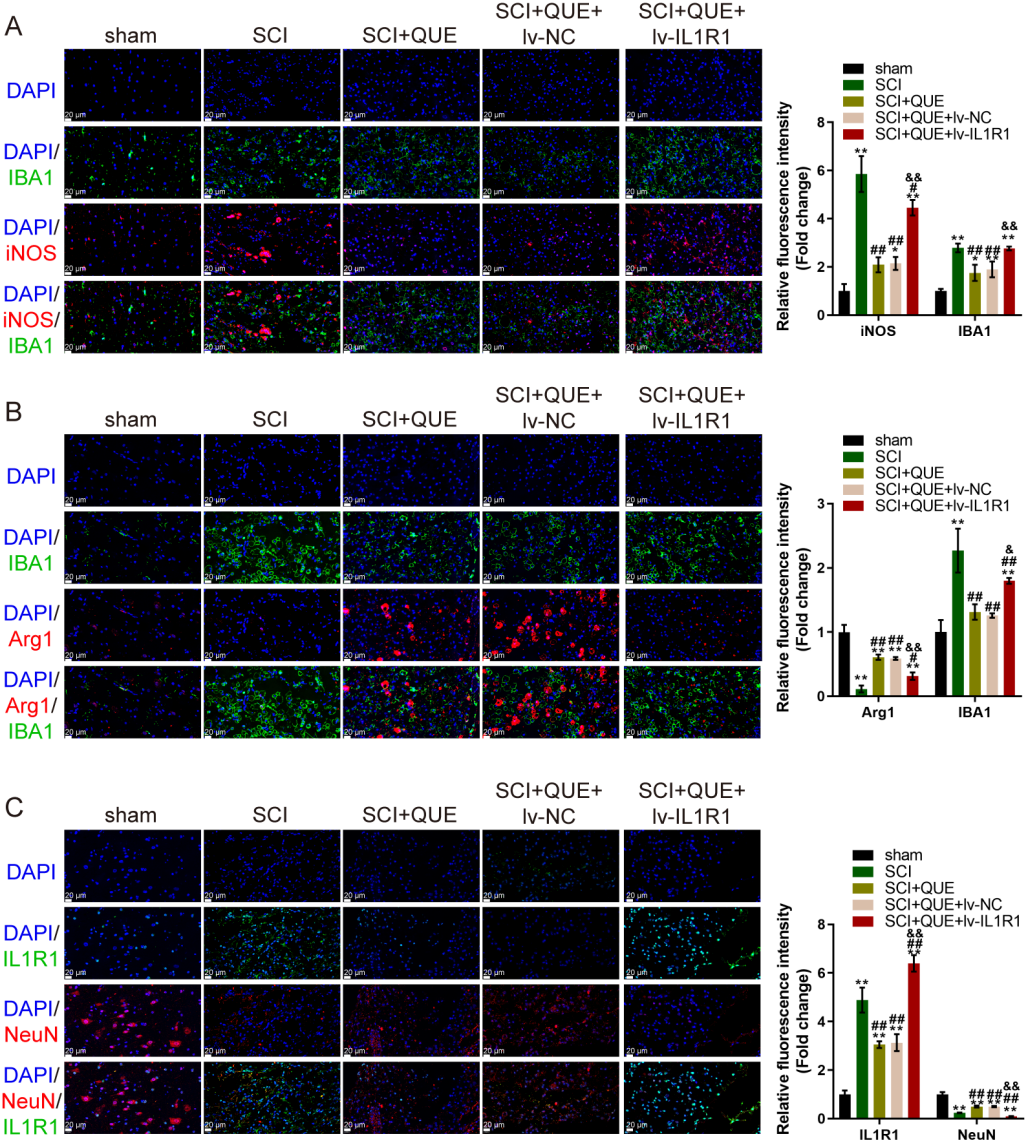
Quercetin modulates microglial polarisation and IL1R1 expression in an SCI rat model. (A) Immunofluorescence (IF staining) images showing DAPI (nuclear marker, blue), IBA1 (activated microglia marker, green), and iNOS (M1 marker, red) in sham, SCI, SCI + QUE, SCI + QUE + lv‐NC, and SCI + QUE + lv‐IL1R1 groups. Quantitative analysis of IBA1/iNOS expression is shown on the right. Scale bar: 20 μm. (B) IF staining images showing DAPI (blue), IBA1 (green), and Arg1 (M2 marker, red) in the same groups. Quantitative analysis of IBA1/Arg1 expression is shown on the right. Scale bar: 20 μm. (C) Co‐localization of IL1R1 (green) and NeuN (neuronal marker, red) in different groups. Quantitative analysis of IL1R1/NeuN co‐localization is shown on the right. Scale bar: 20 μm. * *p* < 0.05, ** *p* < 0.01 represents a comparison with the sham group. ^##^ *p* < 0.01 represents a comparison with the SCI + QUE group. ^&^ *p* < 0.05, ^&&^ *p* < 0.01 represents a comparison with the SCI + QUE + lv‐NC group.

Next, IF staining was performed to assess microglial activation and polarisation in the SCI rat model. IBA1, a marker for activated microglia, was used in combination with iNOS, an M1 polarisation marker. The SCI group exhibited a significant increase in IBA1 and iNOS‐positive cells, indicating heightened pro‐inflammatory M1 microglial activation. However, in the quercetin treatment group (SCI + QUE), iNOS expression was markedly reduced, demonstrating a shift towards an anti‐inflammatory response. In the SCI + QUE + lv‐IL1R1 group, overexpression of IL1R1 partially restored microglia activation and M1 polarisation indicating that IL1R1 overexpression dampens the anti‐inflammatory effects of quercetin (Figure 10A). M2 microglial polarisation was evaluated by staining for Arg1, an M2 marker. In the SCI group, Arg1‐positive cells were significantly lower, reflecting a diminished anti‐inflammatory response. Treatment with quercetin significantly increased Arg1 expression, indicating enhanced M2 polarisation. However, in the SCI + QUE + lv‐IL1R1 group, IL1R1 overexpression reduced the effect of quercetin on promoting M2 polarisation (Figure 10B). In addition, the co‐localisation of IL1R1 (green) and NeuN (red, a neuronal marker) was examined in the SCI rat model. In the SCI group, the number of NeuN‐positive neurons was notably reduced at the injury site, indicating neuronal loss following SCI. Co‐localisation of IL1R1 with NeuN was observed in a small fraction of neurons, indicating that IL1R1 is expressed in microglia and some neurons in the injured spinal cord. After quercetin treatment (SCI + QUE group), the number of NeuN‐positive neurons increased, suggesting a protective effect of quercetin on neuronal survival. Moreover, quercetin significantly reduced IL1R1 expression in both neurons and microglia. In the SCI + QUE + lv‐IL1R1 group, overexpression of IL1R1 restored its expression in neurons and partially reversed the protective effects of quercetin on neuronal survival and IL1R1 suppression (Figure 10C).

## 4 Discussion

A total of 18 active ingredients in 6 herbs of JSK were found to be correlated to inflammation, spinal atrophy and other diseases. These 18 active ingredients target 5464 genes according to the PubChem database. Through comparing differentially expressed genes between SCI and normal samples using datasets GSE42828 and GSE5296, 231 DEGs were identified as overlapping across the datasets and the known drug targets of JSK's active ingredients. Further analysis revealed that 12 of these genes were identified as hub genes. These hub genes were enriched in processes such as oxidative stress response and inflammatory response, consistent with the fact that SCI triggers macrophage/microglia phenotypes that produce oxidant molecules and proinflammatory cytokines [3, 41]. The herb‐compound‐target, herb‐compound‐signalling and compound‐target‐signalling networks were generated using Cytoscape, and quercetin was identified as the hub compound, having the highest degree score among all active compounds, signifying its significant interaction with key target genes. A concentration of 25 μM quercetin showed no cytotoxicity but significantly protected microglial cells from LPS‐induced inhibition of cell viability. LPS stimulation elevated the mRNA levels, culture medium concentrations and protein contents of iNOS, IL‐1β and TNF‐α but decreased IL‐10 levels, whereas 25 μM quercetin significantly attenuated LPS‐induced alterations in these factors. IL1R1 overexpression exerted opposite effects on these factors, and quercetin treatment partially attenuated IL1R1 overexpression‐caused changes in these factors. Furthermore, IL1R1 overexpression promoted the phosphorylation of p38, ERK1/2, JNK and p65 in LPS‐stimulated microglia, whereas quercetin treatment significantly suppressed IL1R1 overexpression‐induced p38, ERK1/2, JNK and p65 phosphorylation protein level. In an SCI rat model, quercetin improved histopathological alterations, local inflammation and facilitated M2 macrophage polarisation at day 14 post‐injury, whereas IL1R1 overexpression partially attenuated the beneficial effects of quercetin on the rat model.

Reportedly, JSK and herbs involved have been widely used in treatment regimens for SCI [12, 14, 15, 16, 17, 42]. In this study, 18 active ingredients were obtained from 6 herbs (OB ≥ 30%, DL ≥ 0.2) according to TCMSP. Obviously, formononetin [43], isorhamnetin [44], calycosin [45], kaempferol [46] and quercetin [47, 48, 49] from HuangQi, Baicalein [50, 51] and Baicalin [52, 53] from ChiShao and HongHua, and beta‐carotene [54], luteolin [55, 56], kaempferol [46] and quercetin [47, 48, 49] from HongHua have been reported to improve inflammation‐ or oxidative stress‐related diseases, including SCI. However, the main active ingredients and hub‐genes dominating JSK protection against SCI and the underlying mechanism remain unclear. Through the PubChem database, a total of 5464 drug target genes were recognised as targets of the 18 active ingredients obtained from JSK's herbs. Among these genes, 231 DEGs were found to overlap with the drug targets and were differentially expressed between SCI and normal samples, with 12 of them identified as hub genes.

Further GO functional enrichment annotation indicated that these 12 hub genes were enriched in oxidative stress response and inflammatory response, which is consistent with the fact that SCI triggers macrophage/microglia phenotypes that produce oxidant molecules and proinflammatory cytokines [3, 41]. Moreover, the herb‐compound‐target, herb‐compound‐signalling and compound‐target‐signalling networks were generated and quercetin was identified as the hub compound. The herb‐compound‐target network, which mapped 50 differentially expressed drug target genes and their related active compounds, revealed the complex interactions between the herbs, active compounds and their target genes. The compound‐signalling network highlighted quercetin's involvement in key pathways, including NF‐κB, PI3K‐Akt, and TNF signalling, all crucial for regulating inflammation and cell survival following SCI [57, 58, 59]. Key targets involved in SCI, including CDK1, FOS, CXCL1, ICAM1, IL1R1, CXCL10, MYC, PTGS2, RB1, CCL2 and TLR4, were significantly upregulated in SCI. Protein structures for these targets were retrieved and docking simulations were performed to assess quercetin's binding interactions with these targets. Among the identified targets, IL1R1 has been recognised as playing a critical role in inflammatory pathways during SCI [37, 38], making it a prime candidate for further experimental validation.

Consistent with the integrative bioinformatics analyses, quercetin has been reported to prevent oligodendrocyte necroptosis through suppressing the polarisation of macrophages and microglia into M1 phenotype following spinal cord injury in rats [47]. In the constructed ingredient‐targets‐signalling pathway network of quercetin, IL1R1 has the greatest number of targeted pathways, including regulation of inflammatory response, NF‐kappa B signalling pathway and MAPK signalling pathway. The inflammation response was shown to be involved. Both neurons [60, 61] and glial cells [62, 63, 64, 65] were shown to express IL1R1, the receptor of IL‐1β in the central nervous system. The prototype of proinflammatory cytokines is IL‐1β which has regulatory effects in various immunological processes [66]. Reportedly, IL‐1β contributes to the pathological mechanism of multiple inflammation diseases that are also linked to pain, including SCI [67]. Thus, quercetin might exert its protective role against SCI in an IL1R1‐related manner.

Highly expressed in SCI, IL1R1 contributes to the inflammatory response after initial injury due to its participation in IL1‐related activities [68]. Although IL1/IL1R1 has been recognised as a double‐edged sword that plays an important role in both exacerbating and mending damage, in SCI, IL1/IL1R1 participates in the initiation and development of inflammation by contributing to the astrocyte‐promoted entrance of inflammatory monocytes and neutrophils [69]. LPS causes morphologic alterations in microglia in the brain and facilitates microglia generation of cytokines and reactive oxygen species [70, 71, 72], and IL‐1/IL1R1 activates astrocytes through actions via IL1R1; both activated microglia and astrocytes may contribute to the secretion of IL‐1 in SCI‐induced neuropathic pain [73, 74, 75]. Therefore, the crosstalk among neurons, microglia and astrocytes promotes neuroinflammation, and this is due in part to IL‐1/IL1R1‐mediated activities [76, 77]. In particular, Bretheau et al. [78] showed that the effects of IL‐1 on neutrophil infiltration following SCI could be completely abrogated by simultaneously injecting IL‐1 and anakinra, an IL‐1 receptor antagonist. In our study, LPS stimulation increased the release of proinflammatory iNOS, IL‐1β and TNF‐α but decreased IL‐10 levels, whereas 25 μM quercetin significantly attenuated LPS‐induced alterations in these factors in microglial cells. In addition to microglia, quercetin has been reported to inhibit astrocyte M1 polarisation [79], reduce neutrophil infiltration [80] and inhibit oligodendrocytes apoptosis [47] during SCI repair. In the meantime, under LPS stimulation, ectopic restoration of IL1R1 significantly attenuated the protective effects of quercetin on microglial cells, suggesting that IL1R1 might participate in quercetin effects on microglia following SCI. As a further confirmation, IL1R1 overexpression dramatically increased the levels of IL‐1β, TNF‐α and iNOS while decreasing IL‐10, which were partially restored by quercetin treatment. Considering that IL‐1β, TNF‐α and iNOS are hallmarks of macrophage M1 polarisation, whereas IL‐10, IL‐10 and low amounts of ROS are those of macrophage M2 polarisation [6, 7], quercetin protection against LPS‐induced damage to microglial cells might be correlated to the regulation of microglial M1/M2 polarisation in an IL1R1 dependent manner. Mechanically, by combining with IL1α or IL1β, IL1R1 upregulation following SCI might activate the NF‐κB signalling pathway, which further upregulates inflammatory genes encoding IL1R1, pro‐IL1α or IL1β and NLRP3 [81]. In our study, IL1R1 overexpression promoted the phosphorylation of p38, ERK1/2, JNK and p65 in LPS‐stimulated microglia, whereas quercetin treatment significantly suppressed IL1R1 overexpression‐induced p38, ERK1/2, JNK and p65 phosphorylation, suggesting the involvement of the NF‐κB and p38 signalling pathways.

More importantly, using an SCI rat model, quercetin improved histopathological alterations, local inflammation and facilitated M2 macrophage polarisation at day 14 post‐injury, whereas IL1R1 overexpression partially attenuated the beneficial effects of quercetin on the rat model, further indicating that quercetin could ameliorate SCI through affecting macrophage polarisation and inflammation post‐injury. During a longer observation period of 28 days after SCI, our findings suggest that both JSK and quercetin provide comparable therapeutic effects in treating SCI, as evidenced by improvements in motor function, downregulation of IL1R1 and modulation of inflammatory cytokines. Importantly, quercetin alone demonstrated therapeutic efficacy similar to that of the complete JSK formulation, indicating that quercetin might be the key active compound responsible for the observed benefits of JSK. However, whether other JSK active ingredients could synergistically interact with quercetin still needs investigation. Additionally, the extended 28‐day observation period revealed that recovery continues beyond 14 days, with sustained improvements in motor function and inflammation, suggesting that both treatments offer long‐lasting benefits.

Collectively, quercetin protection against LPS‐induced cellular inflammation and SCI in rats is likely by modulating microglia polarisation through an IL1R1‐dependent manner. One limitation of our study is the use of a non‐cell‐specific lentiviral vector for IL1R1 overexpression. While this approach successfully increased IL1R1 expression at the injury site, the virus was able to infect multiple cell types including neurons and microglia, potentially confounding the interpretation of our results. Future studies using cell‐type‐specific viral vectors, such as microglia‐specific promoters, would provide more precise insights into the role of IL1R1 in microglial polarisation and quercetin's effects. Besides, the lack of a detailed compositional analysis of the JSK formula should be addressed in future studies. While network pharmacology provided valuable insights into the potential bioactive compounds, including quercetin, we did not quantify the exact concentrations of quercetin in each herb within the JSK formulation. Future studies should focus more on performing a comprehensive chemical analysis to determine the precise levels of quercetin and other key compounds to complement the findings derived from network pharmacology and further elucidate their contributions to the therapeutic effects of JSK.

## Author Contributions

**Lini Dong:** conceptualization (equal), writing – original draft (equal). **Haoyu He:** data curation (equal), methodology (equal). **Zejun Chen:** investigation (equal), software (equal). **Xiaoxiao Wang:** resources (equal), visualization (equal). **Yunchao Li:** formal analysis (equal), validation (equal). **Guohua Lü:** project administration (equal). **Bing Wang:** formal analysis (equal), investigation (equal). **Lei Kuang:** conceptualization (equal), funding acquisition (equal), writing – review and editing (equal).

## Ethics Statement

The authors have nothing to report.

## Conflicts of Interest

The authors declare no conflicts of interest.

## Supporting information


**Figure S1.** Differentially expressed genes between SCI and normal samples according to GSE42828 (A) and GSE5296 (B). (A) Volcano plot showing differentially expressed genes in the GSE42828 dataset. Red dots represent significantly upregulated genes, blue dots represent significantly downregulated genes and grey dots represent genes with |logFC| < 0.6 and adj.*p*.Val < 0.05. The right panel shows a heatmap visualisation of gene expression profiles across the 13 SCI and 4 control mice in GSE42828. (B) Volcano plot showing differentially expressed genes in the GSE5296 dataset. Red, blue and grey dots represent upregulated, downregulated and non‐significant genes, respectively. The right panel shows a heatmap of gene expression profiles across the SCI and sham samples in GSE5296.

**Figure S2.** Quercetin‐target and signalling pathway network generated using Cytoscape software. The network consists of 66 nodes and 251 edges.

**Figure S3.** IL1R1 overexpression was confirmed using qRT‐PCR (A) and Immunoblotting (B). *n* = 3. **p < 0.01 represents a comparison with the lv‐NC group.

**Figure S4.** Comparison of the therapeutic effects of Jisuikang (JSK) and quercetin on SCI recovery at 28 days post‐injury (A) Haematoxylin and eosin (H&E) staining of spinal cord sections from sham, SCI, SCI + JSK and SCI + QUE groups at 28 days post‐SCI. The images show the extent of tissue damage in the SCI group and the improvement in tissue structure with both JSK and QUE treatments. Scale bars: 100 μm (upper row), 50 μm (lower row). (B) BBB scores showing motor function recovery by JSK or quercetin over 28 days post‐SCI. (C) Immunoblotting analysis of IL1R1 protein levels at the injury site treated by JSK or quercetin over 28 days post‐SCI. (D) mRNA expression levels of pro‐inflammatory cytokines (IL‐1β, TNF‐α) and anti‐inflammatory cytokine IL‐10 in the spinal cord treated by JSK or quercetin over 28 days post‐SCI. Data are presented as mean ± SD; *p < 0.05, **p < 0.01 vs. sham group, ^##^ *p* < 0.01 vs. SCI group.

## Data Availability

All the available data were presented in the study.
