# Supplementary figures and images for: Pharmacological Network Analysis of the Functions and Mechanism of Quercetin From Jisuikang (JSK) in Spinal Cord Injury (SCI)
Source: J Cell Mol Med. 2024 Dec 16;28(24):e70269. doi: 10.1111/jcmm.70269 (PMC11648003; doi:10.1111/jcmm.70269)

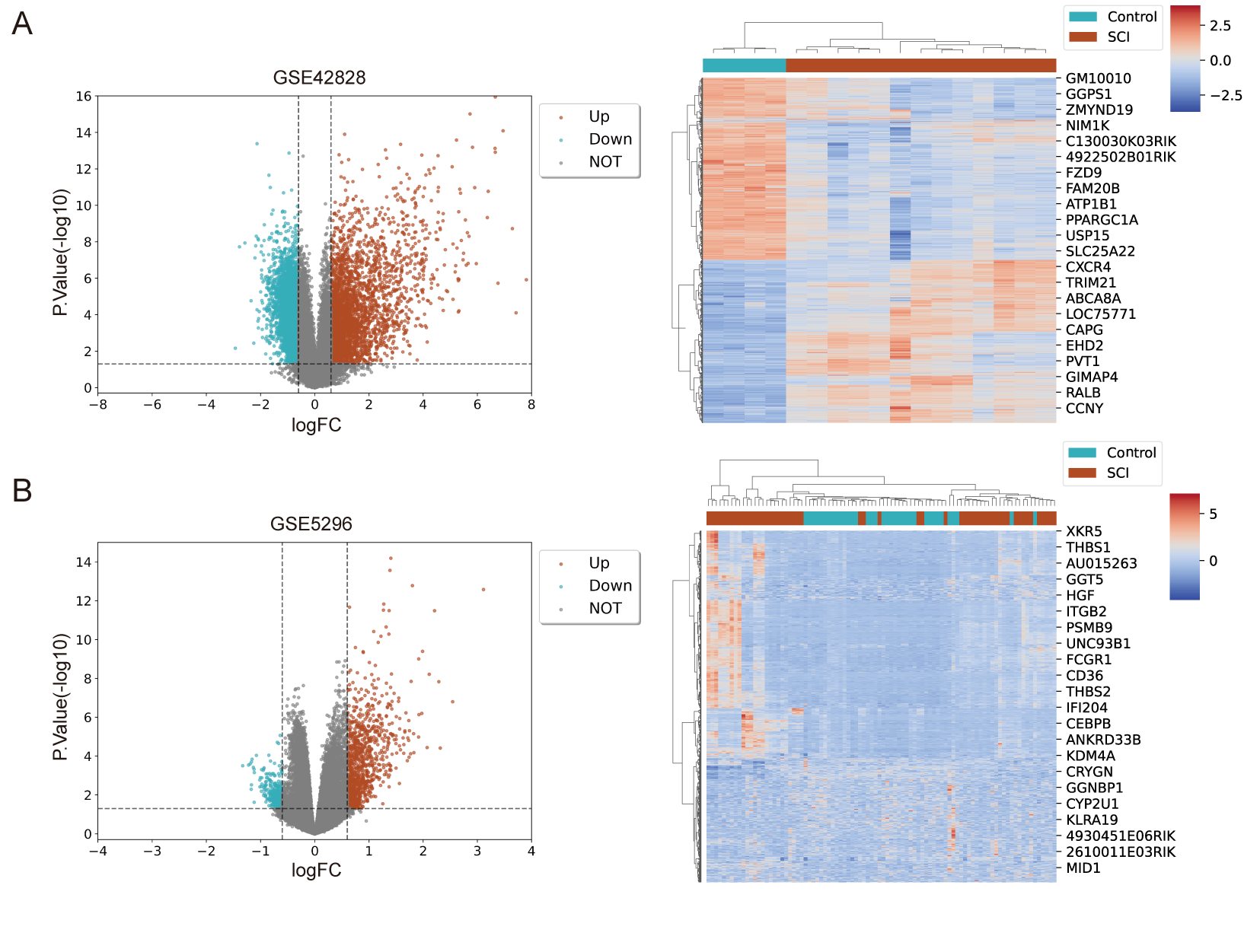

Supplement: Supplementary file 1 — Figure S1. Differentially expressed genes between SCI and normal samples according to GSE42828 (A) and GSE5296 (B). (A) Volcano plot showing differentially expressed genes in the GSE42828 dataset. Red dots represent significantly upregulated genes, blue dots represent significantly downregulated genes and grey dots represent genes with |logFC| < 0.6 and adj.p.Val < 0.05. The right panel shows a heatmap visualisation of gene expression profiles across the 13 SCI and 4 control mice in GSE42828. (B) Volcano plot showing differentially expressed genes in the GSE5296 dataset. Red, blue and grey dots represent upregulated, downregulated and non‐significant genes, respectively. The right panel shows a heatmap of gene expression profiles across the SCI and sham samples in GSE5296. [file JCMM-28-e70269-s003.tif]

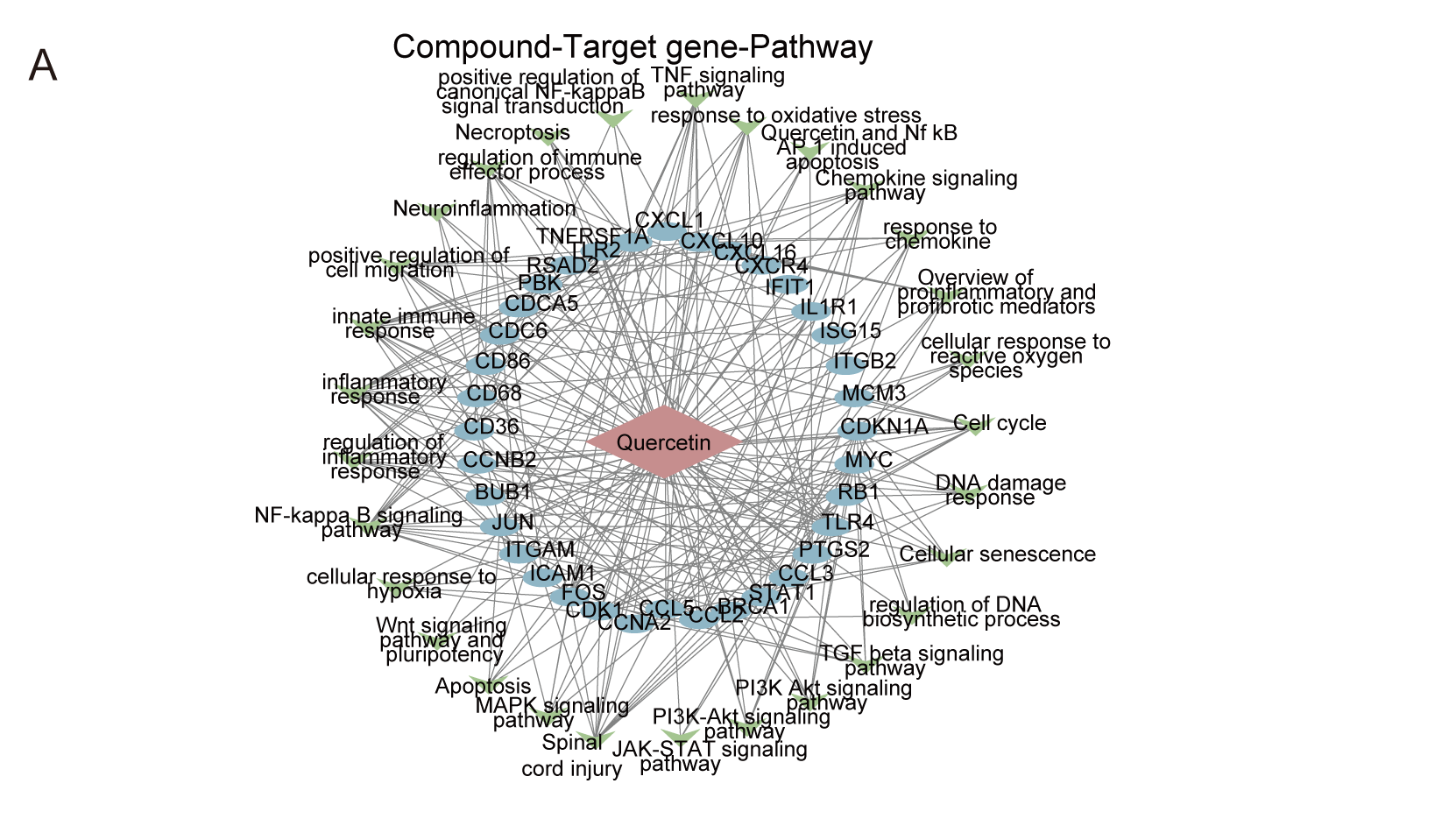

Supplement: Supplementary file 2 — Figure S2. Quercetin‐target and signalling pathway network generated using Cytoscape software. The network consists of 66 nodes and 251 edges. [file JCMM-28-e70269-s002.tif]

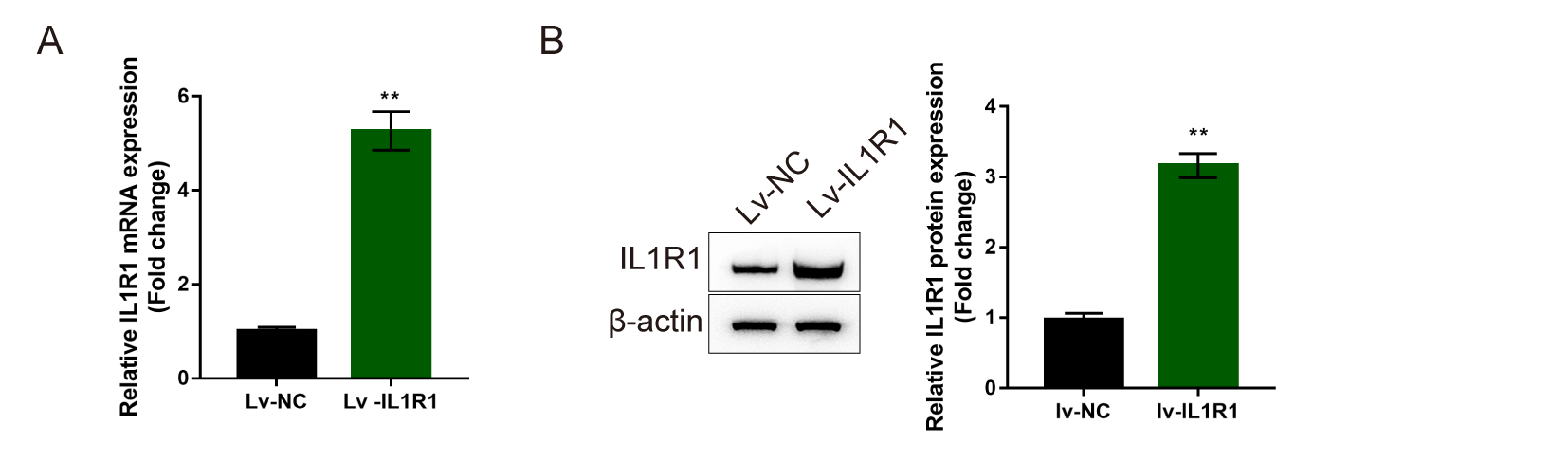

Supplement: Supplementary file 3 — Figure S3. IL1R1 overexpression was confirmed using qRT‐PCR (A) and Immunoblotting (B). n = 3. **p < 0.01 represents a comparison with the lv‐NC group. [file JCMM-28-e70269-s004.tif]

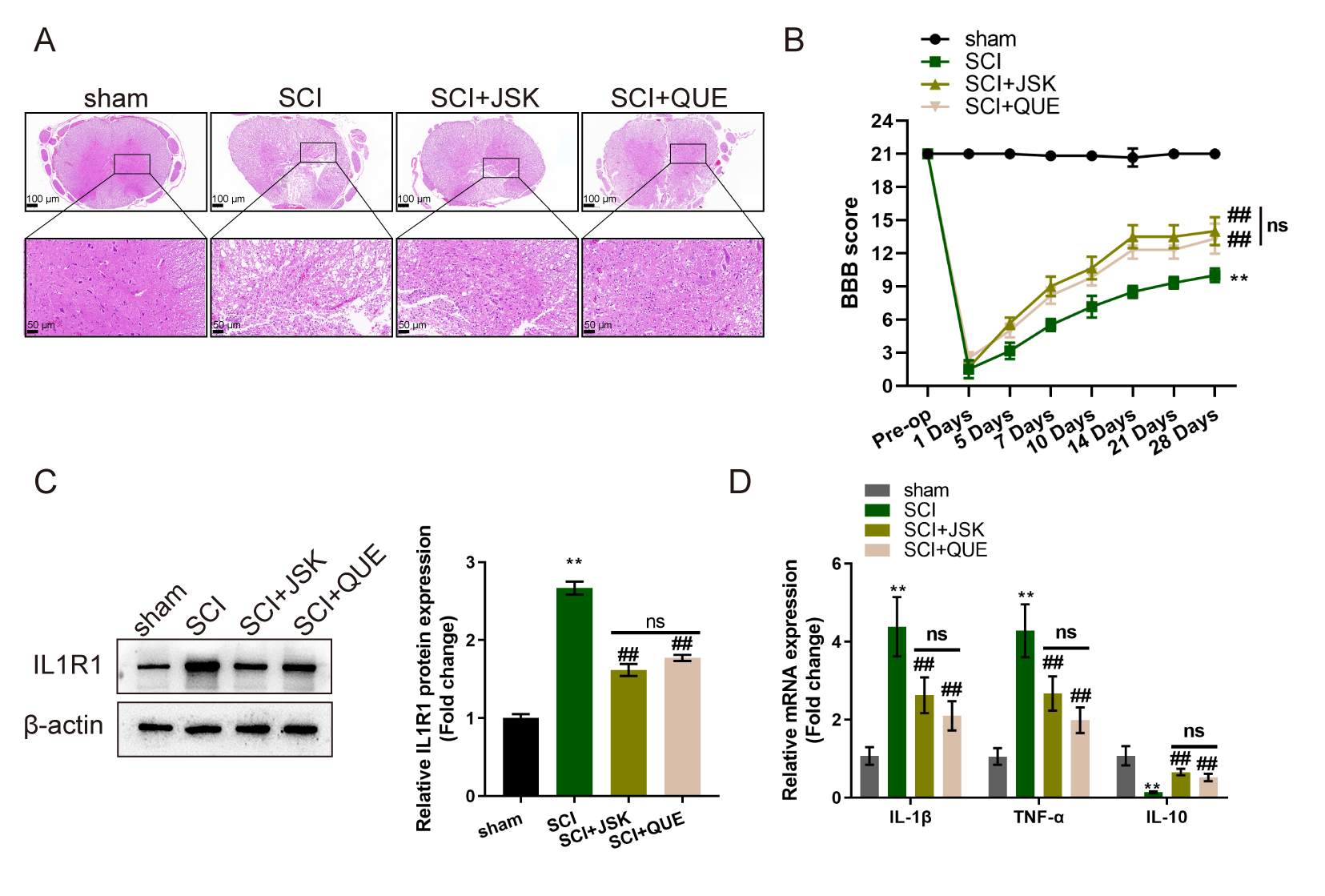

Supplement: Supplementary file 4 — Figure S4. Comparison of the therapeutic effects of Jisuikang (JSK) and quercetin on SCI recovery at 28 days post‐injury (A) Haematoxylin and eosin (H&E) staining of spinal cord sections from sham, SCI, SCI + JSK and SCI + QUE groups at 28 days post‐SCI. The images show the extent of tissue damage in the SCI group and the improvement in tissue structure with both JSK and QUE treatments. Scale bars: 100 μm (upper row), 50 μm (lower row). (B) BBB scores showing motor function recovery by JSK or quercetin over 28 days post‐SCI. (C) Immunoblotting analysis of IL1R1 protein levels at the injury site treated by JSK or quercetin over 28 days post‐SCI. (D) mRNA expression levels of pro‐inflammatory cytokines (IL‐1β, TNF‐α) and anti‐inflammatory cytokine IL‐10 in the spinal cord treated by JSK or quercetin over 28 days post‐SCI. Data are presented as mean ± SD; *p < 0.05, **p < 0.01 vs. sham group, ## p < 0.01 vs. SCI group. [file JCMM-28-e70269-s001.tif]
